# Supplementary material for: Sera Antibody Repertoire Analyses Reveal Mechanisms of Broad and Pandemic Strain Neutralizing Responses after Human Norovirus Vaccination
Source: Immunity. 2019 Jun 18;50(6):1530–1541.e8. doi: 10.1016/j.immuni.2019.05.007 (PMC6591005; doi:10.1016/j.immuni.2019.05.007)
Supplement: Document S1. Figures S1–S3 and Tables S1–S3 [file mmc1.pdf]

Immunity, Volume *50*

Supplemental Information

Sera Antibody Repertoire Analyses Reveal Mechanisms of Broad and Pandemic Strain Neutralizing Responses after Human Norovirus Vaccination

Lisa C. Lindesmith, Jonathan R. McDaniel, Anita Changela, Raffaello Verardi, Scott A. Kerr, Veronica Costantini, Paul D. Brewer-Jensen, Michael L. Mallory, William N. Voss, Daniel R. Boutz, John J. Blazeck, Gregory C. Ippolito, Jan Vinje, Peter D. Kwong, George Georgiou, and Ralph S. Baric

Supplementary Figure Legends

Table S1, related to Figure 1. Description of monoclonal antibodies determined from Donors A, B, and C pre- and post-vaccination.

Clonotype ID	Representative CDR-H3 sequence	SHM (%)	IGHV usage	IGHJ usage	Pre-Vaccination		Post- Vaccination	
					Fraction (%)	XIC	Fraction (%)	XIC
A379	CARDYWNYHIRGWYPDYW	8.6	IGHV3-33	IGHJ4	58.28	5.E+08	31.67	1E+09
A6181	CARADGILVPPRAIWLDHW	12.0	IGHV3-33	IGHJ4	14.79	1.E+08	1.69	6E+07
A12910	CARGNRAVVPSHGFDSW	14.4	IGHV4-31	IGHJ4	6.43	5.E+07	1.67	6E+07
A101	CARSPRTVAIKPYYFDYW	16.7	IGHV3-53	IGHJ4	4.06	3.E+07	10.17	4E+08
A13567	CARLRASGYSYGYGFDHW	6.3	IGHV1-3	IGHJ4	3.96	3.E+07	1.36	5E+07
A1394	CARVPAAIRGVESW	5.2	IGHV1-2	IGHJ5	3.49	3.E+07	0.33	1E+07
A1431	CARDGPRPDGTGYAGPSNDYW	9.7	IGHV3-48	IGHJ4	1.73	1.E+07	10.32	4E+08
A9036	CARGVILARFDSW	4.4	IGHV4-31	IGHJ5	1.59	1.E+07		
A2088	CARLDTYGSGSYYDFW	6.7	IGHV5-51	IGHJ4	1.27	1.E+07		
A15154	CGRDVGPW	7.2	IGHV3-53	IGHJ5	1.13	9.E+06	0.29	1E+07
A11008	CARAGDSSGPW	1.1	IGHV4-34	IGHJ5	0.80	7.E+06	0.08	3E+06
A3865	CAREAIGVSGTTFDLW	17.4	IGHV1-46	IGHJ4	0.73	6.E+06		
A41497	CATLSTRAGGYFDKW	10.3	IGHV4-39	IGHJ4	0.36	3.E+06		
A11742	CARDPPATVNVW	3.3	IGHV3-53	IGHJ6	0.35	3.E+06	0.39	1E+07
A15504	CVRDAYGAFALFDYW	4.8	IGHV3-21	IGHJ4	0.34	3.E+06		
A11065	CARAKGTWGTPLLFW	11.8	IGHV1-69	IGHJ4	0.27	2.E+06		
A6211	CARALNYGDPTALNWFAPW	7.8	IGHV1-69	IGHJ5	0.18	1.E+06		
A1	CASRRRQDTSTVIRLNTMDDGFDVW	6.7	IGHV4-39	IGHJ3	0.12	1.E+06	0.19	7E+06
A12246	CAREGSLRYFDWSRGGTPGYFDYW	1.4	IGHV4-34	IGHJ4	0.10	8.E+05		
A5920	CTRDQCGGDCSDFW	9.3	IGHV3-74	IGHJ4			9.98	4E+08
A834	CARAVPLLGELSIRLSTFDNW	7.8	IGHV4-39	IGHJ4			5.19	2E+08
A4731	CARSRVPSGYCDSVSCYRSYGMDLW	8.9	IGHV1-69	IGHJ6			3.58	1E+08
A16474	CAGRASGSSPPHW	7.9	IGHV3-23	IGHJ4			3.18	1E+08
A16363	CAGHRGYSSGWPLDYW	11.5	IGHV4-34	IGHJ4			2.90	1E+08
A468	CARDRPLASGYDRDDYYGMDVW	7.9	IGHV4-31	IGHJ6			2.26	8E+07
A3612	CARVVAGYYSGSQRYSRSAVIDHW	8.6	IGHV4-59	IGHJ4			1.74	6E+07
A22288	CARAGIIHAINGFDIW	5.8	IGHV1-2	IGHJ3			1.68	6E+07
A10667	CAKGGFDQVPNRENIGGTLLGNW	6.5	IGHV3-23	IGHJ4			1.28	5E+07
A12994	CARGRFLAPFDYW	1.5	IGHV4-34	IGHJ4			1.08	4E+07
A1227	CAKDKTRTLRLGYSGMDVW	8.0	IGHV3-23	IGHJ6			0.90	3E+07
A3804	CARDGALRYDSTGNHYGSW	8.9	IGHV1-2	IGHJ4			0.84	3E+07
A2705	CAKSHYGDTYDSESTYYFDLW	8.8	IGHV1-69	IGHJ2			0.74	3E+07
A3423	CARGTIYDFLAGDRGLGFW	9.3	IGHV1-18	IGHJ4			0.64	2E+07
A4965	CAKCRPGIAAAGPDYW	3.0	IGHV3-30	IGHJ4			0.64	2E+07
A1945	CARGGDYADPQFQRW	5.8	IGHV4-34	IGHJ1			0.52	2E+07
A7704	CVKDRGRRLGSLYPQFGSW	10.5	IGHV3-23	IGHJ4			0.49	2E+07
A2188	CAREGRRDGRPSAVDYW	0.7	IGHV1-2	IGHJ4			0.44	2E+07
A577	CARGSYGDTFGLHPW	8.2	IGHV4-59	IGHJ5			0.43	2E+07
A46215	CATGRVSSNWYRHEYIHYW	7.1	IGHV4-31	IGHJ1			0.40	1E+07
A44984	CARFPSAGYGMDVW	7.6	IGHV3-23	IGHJ6			0.34	1E+07
A4881	CGRWRDGAGAVDNW	7.5	IGHV3-73	IGHJ4			0.33	1E+07
A1438	CARNGEPVYNSWDGNRNHAFDPW	7.8	IGHV4-34	IGHJ3			0.33	1E+07
A5063	CALVTVTRGPVFDYW	5.7	IGHV4-59	IGHJ4			0.28	1E+07
A12572	CARGAYYSDSSGYYKYFHYW	2.6	IGHV3-48	IGHJ1			0.25	9E+06
A3769	CARAGYGSGSYYKHFYYGMDVW	8.5	IGHV5-51	IGHJ6			0.22	8E+06
A2405	CARQQRQSDTSAYYGYW	8.3	IGHV5-51	IGHJ4			0.19	7E+06
A1698	CARGIAARQWYFDLW	4.7	IGHV4-31	IGHJ2			0.19	7E+06
A11278	CARDAGYSSSSENRAPMDW	1.5	IGHV1-2	IGHJ4			0.18	6E+06
A45505	CAQFGSPGLAATLSYFGSW	10.3	IGHV3-23	IGHJ4			0.16	6E+06
A14683	CARYVQGAFDIW	3.0	IGHV4-39	IGHJ3			0.12	4E+06
A28571	CAREKTVSGTDWFDYW	10.8	IGHV4-4	IGHJ4			0.10	4E+06
A14002	CARRVFDHSEYRVAEDKW	9.3	IGHV1-18	IGHJ4			0.08	3E+06
A10111	CVRGIASSVSPFWYFDLW	14.7	IGHV4-39	IGHJ2			0.07	2E+06
A31504	CARGPEKYYDWLGYFDSW	6.3	IGHV4-31	IGHJ4			0.05	2E+06
A3717	CAKDHEVGAPSDLFDYW	6.7	IGHV3-33	IGHJ4			0.03	1E+06
A3439	CARETPGVSDPW	2.5	IGHV4-31	IGHJ5			0.01	4E+05

Clonotype ID	Representative CDR-H3 sequence	SHM (%)	IGHV usage	IGHJ usage	Pre-Vaccination		Post- Vaccination	
					Fraction (%)	XIC	Fraction (%)	XIC
B13309	CAKLDYYDPW	7.6	IGHV3-30	IGHJ5	85.6	1.E+08	13.10	2E+08
B10310	CAKDSGLARSLPDYW	4.0	IGHV3-9	IGHJ4	4.20	5.E+06		
B3312	CARRDGVLDW	1.2	IGHV1-46	IGHJ4	3.12	4.E+06		
B9951	CSRDVARARRFGELRTLSDYASLPSSKPFDPW	13.4	IGHV3-49	IGHJ5	3.00	4.E+06		
B6525	CARAGGSGTYYPNWFDPW	7.2	IGHV4-39	IGHJ5	2.88	3.E+06		
B5394	CARALFSNYYDNRGYYHELGHW	8.6	IGHV3-30	IGHJ4	1.24	1.E+06		
B10587	CAKVRGRVGGATFYHGMDVW	10.3	IGHV3-23	IGHJ6			47.50	6E+08
B231	CARLKSAYYFDSW	12.5	IGHV1-2	IGHJ4			8.23	1E+08
B401	CSRGKRRVAGPTFYSGMDVW	11.8	IGHV3-23	IGHJ6			6.77	9E+07
B137	CARMASGYYDWW	7.1	IGHV1-2	IGHJ4			5.15	7E+07
B12268	CARVLETNWDPNWFDRW	7.1	IGHV4-31	IGHJ5			4.17	6E+07
B403	CARGEGGGTLPFEYW	9.2	IGHV4-31	IGHJ4			3.97	5E+07
B3742	CARHFGSGSGSYSRTVQSW	4.3	IGHV4-39	IGHJ5			2.29	3E+07
B404	CAKGKRRVGGAAFYSGLDVW	9.0	IGHV3-23	IGHJ6			2.00	3E+07
B6907	CARVMEQYYDSRAMDHW	8.2	IGHV4-31	IGHJ4			1.15	2E+07
B4651	CARFDCFSTTCRHYYAMDVW	7.7	IGHV4-39	IGHJ6			1.02	1E+07
B5944	CARDFSDW	4.4	IGHV4-39	IGHJ4			0.99	1E+07
B68	CARDRGGRDLDTW	8.1	IGHV3-33	IGHJ5			0.97	1E+07
B542	CARLLYDRSGYYYFDSW	4.9	IGHV4-39	IGHJ4			0.92	1E+07
B1021	CARDPGGVLVAGLFHRNDVCDIW	10.9	IGHV1-3	IGHJ3			0.80	1E+07
B14504	CARDRGFYGVDVW	13.2	IGHV1-3	IGHJ6			0.57	8E+06
B980	CARVGLMGGSTPFDYW	3.6	IGHV4-31	IGHJ4			0.16	2E+06
B11062	CAREVSTGACGYW	0.0	IGHV3-7	IGHJ4			0.14	2E+06
B3645	CARDDVRYGFMVYW	8.7	IGHV1-18	IGHJ4			0.11	1E+06
B44	CARGGYGANYGDHW	10.9	IGHV7-81	IGHJ4			0.01	1E+05
C601	CARAAPLQWELKPGPFNSW	7.5	IGHV3-53	IGHJ4	70.36	8E+08	91.92	1E+10
C16720	CSRIGFLQWSRSNDAFDVW	15.1	IGHV4-55	IGHJ3	8.17	9E+07	2.05	3E+08
C46	CARDLKFGVGVPLTPYYDSW	10.8	IGHV3-11	IGHJ4	7.21	8E+07	0.75	9E+07
C26886	CAKDRNTNRDGLGNHFDSW	8.9	IGHV1-2	IGHJ5	6.05	7E+07	1.38	2E+08
C1768	CARLRVGSSSGRELDYW	4.0	IGHV5-51	IGHJ4	3.80	4E+07	0.23	3E+07
C159	CAKVAGTSFYYFYAMDVW	4.3	IGHV3-30	IGHJ6	1.36	2E+07	0.05	6E+06
C21286	CARPAAGALRTGDYSYGFDPW	15.0	IGHV1-18	IGHJ5	1.17	1E+07		
C10582	CARGNQGVELRKFWFDPW	11.9	IGHV4-59	IGHJ5	0.69	8E+06	0.49	6E+07
C13227	CARWDPYDFW	13.3	IGHV1-3	IGHJ4	0.42	5E+06		
C12556	CARGPRRIMHYYDNRGPIDFW	12.2	IGHV3-30	IGHJ4	0.38	4E+06		
C9325	CARRGVRIPLDAFDVW	12.8	IGHV4-39	IGHJ3	0.37	4E+06		
C602	CARGRSGWETRPHYFDYW	7.9	IGHV3-49	IGHJ4			1.11	1E+08
C12468	CARGGWSMDVW	6.6	IGHV4-59	IGHJ6			0.65	8E+07
C380	CAKAKGDYYESSGYLFDSW	3.4	IGHV3-23	IGHJ4			0.54	7E+07
C12232	CAREGGGYFYGPGNHYRYYYAMDVW	10.8	IGHV4-59	IGHJ6			0.27	3E+07
C9502	CARVSRTTWGFGYW	4.4	IGHV4-31	IGHJ4			0.17	2E+07
C39728	CARGRPPNWELFVDYFDYW	8.4	IGHV3-53	IGHJ4			0.16	2E+07
C6265	CARVFLDYEIRTGYYTQYNFDYW	9.5	IGHV4-39	IGHJ4			0.10	1E+07
C2873	CARSPPNTNYLRDAFDIW	13.4	IGHV4-59	IGHJ3			0.09	1E+07
C7027	CARRGSRTAASYGMDVW	0.8	IGHV1-2	IGHJ6			0.03	4E+06

Table S2, related to Figures 4 and 5. Crystallographic data collection and refinement statistics.

	**GII.4.2002+A1431**	**GII.4.2002+A1227**
PDB ID	6N8D	6N81
**Data collection**		
Space group	C 2	P 2_1
Cell dimensions		
a, *b*, *c* (Å)	147.4, 157.4, 110.4	82.6, 150.1, 117.3
α, β, γ (°)	90, 112.9, 90	90, 95.1, 90
Resolution (Å)	50 - 3.1 (3.15-3.10)*	50 – 2.6 (2.64-2.60)
Unique reflections	39748 (2068)	84782 (4171)
R_{merge}	13.6 (87.7)	12.0 (45.3)
R_{pim}	8.5 (55.4)	7.8 (30.7)
$I / \sigma I$	15.7 (2.2)	12.3 (2.1)
Completeness (%)	94.9 (97.4)	96.0 (95.5)
Redundancy	3.4 (3.4)	3.2 (2.8)
Wilson B-factors (Å^2)	61	31
**Refinement**		
Resolution (Å)	41.62 – 3.10 (3.21-3.10)	46.09 - 2.58 (2.67 – 2.58)
No. reflections	39673 (3942)	84760 (7381)
R_{work} / R_{free}	0.18 (0.26) / 0.23 (0.33)	0.17 (0.24) / 0.21 (0.30)
No. atoms	11214	11539
Protein	11214	11232
Water	0	307
B-factors (Å^2)		
P domain	49	38
Fab	109	54
Water	-	39
R.m.s. deviations		
Bond lengths (Å)	0.007	0.003
Bond angles (°)	1.26	0.78
Ramachandran statistics		
Favored (%)	92	96
Outliers (%)	0.6	0.3

One crystal was used for each structure

* Values in parentheses are for highest-resolution shell

Table S3, related to STAR methods. Primers for amplifying multiplex VH repertoire.

V_H	Primer (5'->3')
VH1-fwd	CAGGTCCAGCTKGTRCAGTCTGG
VH157-fwd	CAGGTGCAGCTGGTGSARTCTGG
VH2-fwd	CAGRTCACCTTGAAGGAGTCTG
VH3-fwd	GAGGTGCAGCTGKTGGAGWCY
VH4-fwd	CAGGTGCAGCTGCAGGAGTCSG
VH4-DP63-fwd	CAGGTGCAGCTACAGCAGTGGG
VH6-fwd	CAGGTACAGCTGCAGCAGTCA
VH3N-fwd	TCAACACAACGGTTCCCAGTTA
IgM-rev	GGTTGGGGCGGATGCACTCC
IgG-all-rev	SGATGGGCCCTTGGTGGARGC
IgA-all-rev	GGCTCCTGGGGGAAGAAGCC

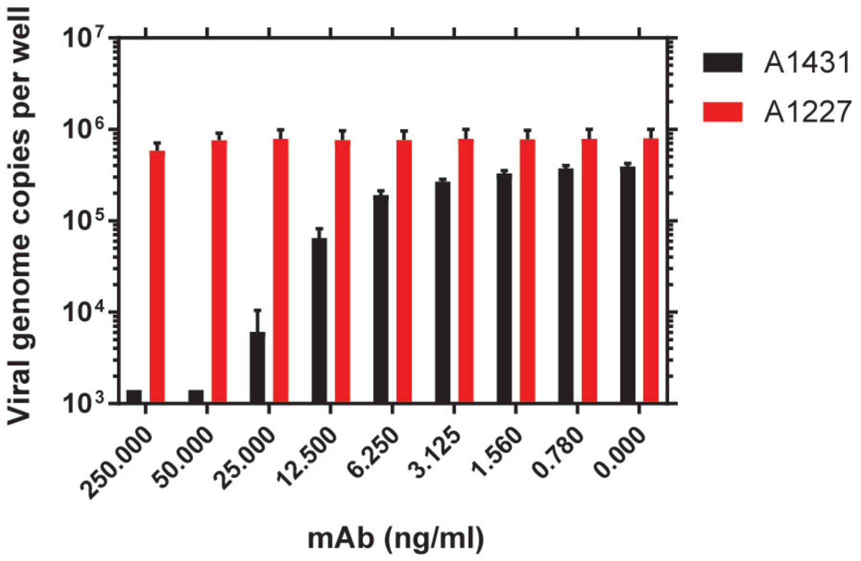

Figure S1, related to Figure 2. A1431 neutralizes GII.P16-GII.4 Sydney strain.

Viral genome copies per well at 24hs post-infection as a function of antibody concentration. Error bars represent SEM. Samples were tested in duplicate in four independent experiments.

a

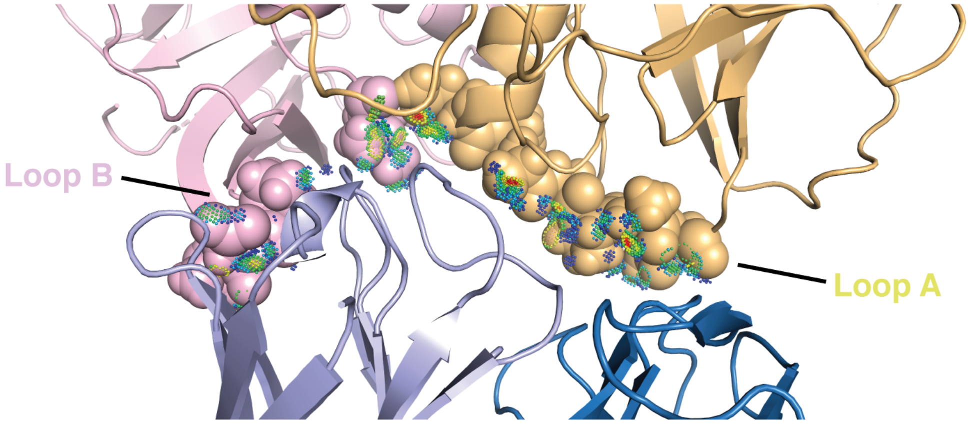

b

```
         448       458       468       478       488       498
GII.4    NLDCLLPQEWVQHFYQEAAPAQSDVALLRFVNPDTGRVLFECKLHKSGYVTVAHT
GII.14   AIDCLLPQEWIEHFYQEAAPSQSDIALVRFINPDTGRVLLEAKLHKQGFLTVAAS
GII.3    VLDCLVPQEWVQHFYQESAPAQTQVALVRYVNPDTGRVLFEAKLHKLGFMTIAKN
GII.17   IIDCLIPQEWIQHFYQESAPSQSDVALIRYVNPDTGRTLFEAKLHRSGYITVAHS
GII.2    AIDCLLPQEWVQHFYQEAAPSMSEVALVRYINPDTGRALFEAKLHRAGFMTVSSN
GII.1    AIDCLLPQEWIQHFYQESAPSPTDVALIRYTNPDTGRVLFEAKLHRQGFITVANS
GI.3     SVPCTIPQEFVTHFVNEQAPTRGEAALLHYLDPDTHRNLGEFKLYPEGFMTCVPN
GI.1     NLPCLLPQEYISHLASEQAPTVGEAALLHYVDPDTGRNLGEFKAYPDGFLTCVPN
G1.4     LVPCLLPQEYITHFISEQAPIQGEAALLHYVDPDTNRNLGEFKLYPGGYLTCVPN
                         Loop A               Loop B
```

Figure S2, related to Figure 4. A1227 interacts with the GII.4.2002 P domain. **a**) Two main loops in P2002 are responsible for interaction with A1227. Loop A (orange spheres) is composed of 7 amino acids (Q463-Q469) and is located at the C-terminus of the A monomer. Loop B (pink spheres) is composed of 3 amino acids (D481-G483) and is located at the C-terminus of monomer B. van der Waals (vdW) interactions were calculated using Protein-Interaction-Viewer software installed as Pymol plugin (Word et al., 1999), which implements the small-probe contact dot surfaces technique. Dot colors indicate the gap distance between vdW radii at each dot position (using a 0.25 Å probe): green or yellow for good contact (greens for narrow gaps, yellows for slight overlaps <0.2 Å), blues for wider gaps (>0.25 Å), orange or red spikes for small interpenetrations. **b**) Sequence alignment of several GI and GII genotypes highlighting degree of conservation among interacting residues (Deep purple denotes identical residues).

```
GII.4.2002       AHQNEPQQWVLPSYSGRTGHNVHLAPAVAPTFPGEQLLFFRSTMPGCSGYPNM-NLDCLL   453
GII.4.1987       HHQNEPQQWVLPNYSGRTGHNVHLAPAVAPTFPGEQLLFFRSTMPGCSGYPNM-NLDCLL   452
GII.4.1997       NHQNEPQQWVLPNYSGRTGHNVHLAPAVAPTFPGEQLLFFRSTMPGCSGYPNM-NLDCLL   452
GII.4.2006a      THQNEPQQWELPDYSGRGIHNVHLAPAVAPTYPGEQILFFRSTMPGCSGYPNM-NLDCLL   453
GII.4.2006b      THRNEPQQWVLPSYSGRNVHNVHLAPAVAPTFPGEQLLFFRSTMPGCSGYPNM-DLDCLL   453
GII.4.2012       THRNEPQQWVLPSYSGRNTHNVHLAPAVAPTFPGEQLLFFRSTMPGCSGYPNM-DLDCLL   453
GII.4.2015       THRNEPQQWVLPSYSGRNTHNVHLAPAVAPTFPGEQLLFFRSTMPGCSGYPNM-DLDCLL   453
GI.1             PSGSQVDLWKIPNYGSSITEATHLAPSVYPPGFGEVLVFFMSKMPGPGAY----NLPCLL   437
GI.3             GHRGSVDPWVIPRYGSTLTEAAQLAPPIYPPGFGEAIVFFMSDFPIAHGA-NGLSVPCTI   452
GI.4             SGGANTNFWKIPDYGSSLAEASQLAPAVYPPGFNEVIVYFMASIPGPNQSGSPNLVPCLL   451
GII.1            ----HFDQWALPSYSGRLTLNMNLAPSVSPLFPGEQLLFFRSHIPLKGGTSDG-AIDCLL   448
GII.2            ----HFNQWVVPRYAGALNLNTNLAPSVAPVFPGERLLFFRSYIPLKGGYGNP-AIDCLL   455
GII.3            --EAEFQQWSLPNYSGQFTHNMNLAPAVAPNFPGEQLLFFRSQLPSSGGWSNG-VLDCLV   461
GII.14           --QHPFRQWSLPNYGGHLALNNHLAPAVTPLFPGEQILFFRSHIPSAGGHTDG-AIDCLL   449
GII.17           -DGHPFRQWELPNYSGELTLNMNLAPPVAPNFPGEQLLFFRSFVPCSGGYNQG-IIDCLI   453
                         * :* *..      :*** : *   .* :::* : .*        : * :

GII.4.2002       PQEWVQHFYQEAAPAQSDVALLRFVNPDTGRVLFECKLHKSGYVTVAHT--GQHDLVIPP   511
GII.4.1987       PQEWVLHFYQEAAPAQSDVALLRFVNPDTGRVLFECKLHKSGYITVAHT--GPYDLVIPP   510
GII.4.1997       PQEWVQHFYQEAAPAQSDVALLRFVNPDTGRVLFECKLHKSGYVTVAHT--GPHDLVIPP   510
GII.4.2006a      PQDWVRHFYQEAAPAQSDVALLRFVNPDTGRVLFECKLHKSGYVTVAHT--GQHDLVIPP   511
GII.4.2006b      PQEWVQHFYQEAAPAQSDVALLRFVNPDTGRVLFECKLHKSGYVTVAHT--GQHDLVIPP   511
GII.4.2012       PQEWVQYFYQEAAPAQSDVALLRFVNPDTGRVLFECKLHKSGYVTVAHT--GQHDLVIPP   511
GII.4.2015       PQEWVQYFYQEAAPAQSDVALLRFVNPDTGRVLFECKLHKSGYVTVAHT--GQHDLVIPP   511
GI.1             PQEYISHLASEQAPTVGEAALLHYVDPDTGRNLGEFKAYPDGFLTCVPNGASSGPQQLPI   497
GI.3             PQEFVTHFVNEQAPTRGEAALLHYLDPDTHRNLGEFKLYPEGFMTCVPNSSGTGPQTLPI   512
GI.4             PQEYITHFISEQAPIQGEAALLHYVDPDTNRNLGEFKLYPGGYLTCVPNSSSTGPQQLPL   511
GII.1            PQEWIQHFYQESAPSPTDVALIRYTNPDTGRVLFEAKLHRQGFITVANS--GSRPIVVPP   506
GII.2            PQEWVQHFYQEAAPSMSEVALVRYINPDTGRALFEAKLHRAGFVTVSSN--TSAPVVVPA   513
GII.3            PQEWVQHFYQESAPAQTQVALVRYVNPDTGRVLFEAKLHKLGFMTIAKN--GDSPITVPP   519
GII.14           PQEWIEHFYQEAAPSQSDIALVRFINPDTGRVLLEAKLHKQGFLTVAAS--GDHPIVMPT   507
GII.17           PQEWIQHFYQESAPSQSDVALIRYVNPDTGRTLFEAKLHRSGYITVAHS--GDYPLVVPA   511
                 **::: :: .* **   : **::: :*** * * * * :  *::*   .        :*
```

Figure S3, related to Figure 5. A ClustalW alignment of GI and GII P-domain sequences shows residues that directly interact with A1431 are conserved among GII.4 strains but differ in other GI and GII strains. Consensus symbols indicate varying degrees of conservation at each position: Fully conserved (*), strong (:) and weak (.) level of conservation. Residues involved in binding A1431 Fab are highlighted in blue (see **Fig. 5c** for interaction with A1431 and **Fig. 5d** for location of these residues on the P domain dimer).
