# Supplementary material for: Sera Antibody Repertoire Analyses Reveal Mechanisms of Broad and Pandemic Strain Neutralizing Responses after Human Norovirus Vaccination
Source: Immunity. 2019 Jun 18;50(6):1530–1541.e8. doi: 10.1016/j.immuni.2019.05.007 (PMC6591005; doi:10.1016/j.immuni.2019.05.007)
Supplement: Document S2. Article plus Supplemental Information [file mmc2.pdf]

Immunity

Article

Sera Antibody Repertoire Analyses Reveal Mechanisms of Broad and Pandemic Strain Neutralizing Responses after Human Norovirus Vaccination

Graphical Abstract

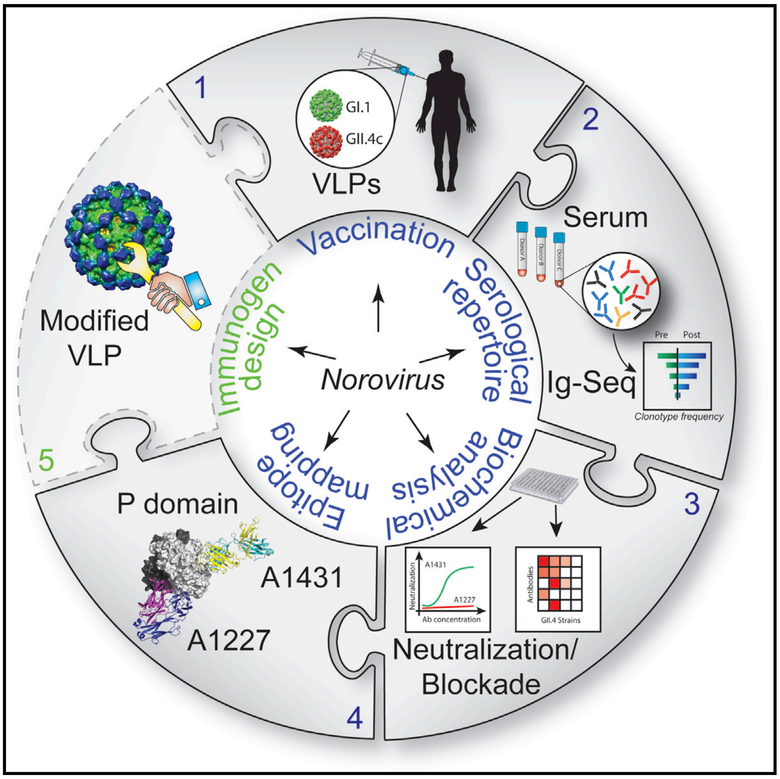

Highlights

- Serum vaccine response is dominated by a small number of abundant antibody clonotypes
- Vaccine-boosted antibodies predominantly target conserved norovirus epitopes
- Identified cross-genogroup and strain-specific epitopes
- Discovered a pandemic-genotype neutralizing antibody recognizing a conserved epitope

Authors

Lisa C. Lindesmith,
Jonathan R. McDaniel,
Anita Changela, ..., Peter D. Kwong,
George Georgiou, Ralph S. Baric

Correspondence

gg@che.utexas.edu (G.G.),
rbaric@email.unc.edu (R.S.B.)

In Brief

Human norovirus (HuNoV) is a leading cause of gastroenteritis. Lindesmith et al. identify circulating serum antibodies following experimental HuNoV vaccination in humans and map them to viral epitopes. One antibody recognizes a neutralizing epitope conserved across three decades of pandemic strains and neutralizes virus *in vitro*, demonstrating that vaccination can elicit pandemic-strain neutralizing antibody responses in some individuals.

Check for updates

Lindesmith et al., 2019, Immunity *50*, 1530–1541
June 18, 2019 © 2019 The Author(s). Published by Elsevier Inc.
https://doi.org/10.1016/j.immuni.2019.05.007

CellPress

Immunity
Article

Sera Antibody Repertoire Analyses Reveal Mechanisms of Broad and Pandemic Strain Neutralizing Responses after Human Norovirus Vaccination

Lisa C. Lindesmith,[1,7] Jonathan R. McDaniel,[2,7] Anita Changela,[3,7] Raffaello Verardi,[3,7] Scott A. Kerr,[4,7,9] Veronica Costantini,[6] Paul D. Brewer-Jensen,[1] Michael L. Mallory,[1] William N. Voss,[2] Daniel R. Boutz,[4] John J. Blazeck,[2] Gregory C. Ippolito,[5] Jan Vinje,[6] Peter D. Kwong,[3] George Georgiou,[2,4,5,8,*] and Ralph S. Baric[1,8,10,*]
[1]Department of Epidemiology, University of North Carolina, Chapel Hill, NC 27599, USA
[2]Department of Chemical Engineering, University of Texas at Austin, Austin, TX 78712, USA
[3]Vaccine Research Center, National Institute of Allergy and Infectious Diseases, National Institutes of Health, Bethesda, MD 20892, USA
[4]Institute for Cellular and Molecular Biology, University of Texas at Austin, Austin, TX 78712, USA
[5]Department of Molecular Biosciences, University of Texas at Austin, Austin, TX 78712, USA
[6]Division of Viral Diseases, Centers for Disease Control and Prevention, Atlanta, GA 30333, USA
[7]These authors contributed equally
[8]These authors contributed equally
[9]Present address: BioFire Inc, Salt Lake City, UT 84108, USA
[10]Lead Contact
*Correspondence: gg@che.utexas.edu (G.G.), rbaric@email.unc.edu (R.S.B.)
https://doi.org/10.1016/j.immuni.2019.05.007

SUMMARY

Rapidly evolving RNA viruses, such as the GII.4 strain of human norovirus (HuNoV), and their vaccines elicit complex serological responses associated with previous exposure. Specific correlates of protection, moreover, remain poorly understood. Here, we report the GII.4-serological antibody repertoire—pre- and post-vaccination—and select several antibody clonotypes for epitope and structural analysis. The humoral response was dominated by GII.4-specific antibodies that blocked ancestral strains or by antibodies that bound to divergent genotypes and did not block viral-entry-ligand interactions. However, one antibody, A1431, showed broad blockade toward tested GII.4 strains and neutralized the pandemic GII.P16-GII.4 Sydney strain. Structural mapping revealed conserved epitopes, which were occluded on the virion or partially exposed, allowing for broad blockade with neutralizing activity. Overall, our results provide high-resolution molecular information on humoral immune responses after HuNoV vaccination and demonstrate that infection-derived and vaccine-elicited antibodies can exhibit broad blockade and neutralization against this prevalent human pathogen.

INTRODUCTION

Human norovirus (HuNoV) is the leading cause of acute gastroenteritis, a disease responsible for an estimated 200,000 deaths per year, mostly in children less than five years old and the elderly (Collaborators, 2017; Patel et al., 2008). The human norovirus genome is a single-stranded, plus-sensed RNA with 3 open reading frames (ORFs), including an ORF2 major capsid protein (viral protein 1, VP1) composed of shell and protruding (P) domains. Expression of VP1 in insect and mammalian systems leads to self-assembly of 90 VP1 dimers into icosahedral virus-like particles (VLPs) that are antigenically indistinguishable from virions (Baric et al., 2002; Green et al., 1993; Jiang et al., 1995; Prasad et al., 1999). Despite more than 30 known human norovirus genotypes, ~60% of norovirus outbreaks are caused by GII.4 genotype strains (Burke et al., 2019; Cannon et al., 2017). Efforts to develop an effective vaccine against human norovirus are complicated by the large number of antigenically distinct genotypes and the rapid evolution of GII.4 viruses (Atmar et al., 2018; Bull et al., 2007; Debbink et al., 2014a; Kocher et al., 2018; Lindesmith et al., 2008). Increased understanding of which capsid epitopes should be targeted to protect against different genotypes and of the impact of pre-exposure history on the elicitation of protective immunity after vaccination are important considerations in vaccine design and efficacy (Havenar-Daughton et al., 2018; Lindesmith et al., 2015, 2017; Snijder et al., 2018).

Antigenic drift within GII.4 norovirus strains has prompted serial pandemic outbreaks in 1995, 2002, 2006, 2009, and 2012, each correlating with the emergence of new GII.4 antigenic variants that appeared to have escaped pre-existing immunity (Debbink et al., 2012b; Lindesmith et al., 2008; Siebenga et al., 2007). Human norovirus attaches to the cell surface via binding to histoblood group antigens (HBGAs; typically A, B, O, or Lewis antigens) on mucosal surfaces of the gut (Lindesmith et al., 2003; Tan and Jiang, 2011). Extensive studies have established that antibodies that block norovirus VLP binding to HBGAs strongly correlate with protective immunity in chimpanzees and humans (Atmar et al., 2011; Bok et al., 2011; Malm et al., 2014; Reeck et al., 2010). Immune escape frequently relies on mutations at key residues in the immunodominant A, D, and E epitopes targeted by blockade

 © 2019 The Author(s). Published by Elsevier Inc.
This is an open access article under the CC BY license (http://creativecommons.org/licenses/by/4.0/).

antibodies, with blockade measured by a surrogate neutralization assay based on the antibody-dependent inhibition of VLP binding to HBGAs (Debbink et al., 2012a; Harrington et al., 2002; Lindesmith et al., 2012a, 2012b). Carbohydrate ligand blockade assays are the primary functional assay used to predict serological immunity and represent the clinical endpoint for evaluating norovirus infection and vaccination outcomes (Atmar et al., 2011; Lindesmith et al., 2015, 2017; Malm et al., 2014; Reeck et al., 2010). Recent development of human intestinal enteroids (HIEs) as an *in vitro* system that successfully supports the replication of HuNoV also allows for direct evaluation of neutralization (Costantini et al., 2018; Ettayebi et al., 2016).

Multivalent VLP immunization broadens the breadth of blockade antibodies produced after immunization of mice (Debbink et al., 2014b; LoBue et al., 2006) and humans (Lindesmith et al., 2015). Currently, the leading human norovirus vaccine candidate is in phase IIb clinical trials (Bernstein et al., 2015; Leroux-Roels et al., 2018). This vaccine comprises a mixture of two VLPs: GI.1, the prototypical genogroup 1 strain, and GII.4c (GII.4 consensus), a VLP based on the consensus sequence of the GII.4 strains Houston (2002), Yerseke (2006a), and Den Haag (2006b) (Bernstein et al., 2015; Parra et al., 2012; Treanor et al., 2014). GII.4.2006a was used as the default sequence where all three of the strains diverged, most notably within the evolving immunodominant epitopes A, D, and E. The vaccine induces rapid (evident at day 7 post vaccination) antibody responses against both genogroup I and genogroup II VLPs (Lindesmith et al., 2015). The rapid blockade antibody response after vaccination suggests that the vaccine may activate a memory B cell or recall response in adults (Lindesmith et al., 2015), though the mechanisms governing this immune outcome have remained unknown.

Here, we report on the serological repertoire to the GII.4c VLP component of the bivalent human norovirus vaccine pre- and post-immunization. We determined the serological repertoire using immunoglobulin sequencing (Ig-Seq), a proteomics-based serum antibody repertoire analysis methodology (Georgiou et al., 2014; Lee et al., 2016; Williams et al., 2017). We focused on the GII.4c component of the vaccine because GII.4 strains have proven more clinically significant and display a higher rate of evolutionary drift, which potentially complicates the elicitation of protective immunity. Our results (1) provide clear evidence of the dominant effect of pre-existing immunity arising from earlier exposure on the humoral response to the vaccine; (2) define three classes of HuNoV circulating antibodies: one class comprising antibodies with very extensive binding breadth recognizing GI strains and GII strains but having no blockade activity, a second class that is specific to GII.4 and has blockade activity towards historical pandemic strains, and finally, a third class represented by one virus-neutralizing antibody with potent blockade activity towards historical GII.4 strains and contemporary strains that emerged well after the strains which were used to design the vaccine GII.4c VLP and human trials; (3) characterize in molecular detail VP1 epitopes that are targeted to enable broad binding or broad blockade with neutralizing activity, and (4) highlight the impact of pre-existing serological repertoire breadth and titers on the response to the vaccine.

RESULTS

The GII.4 HuNoV Serological Repertoire after HuNoV Bivalent Vaccination Is Highly Polarized and Shaped by Previous GII.4 Infection

We analyzed three donors that experienced a significant increase in GII.4 titer after immunization with the bivalent GII.4c + GI.1 VLP vaccine (Figure 1A; Table S1). The serological repertoire was delineated using Ig-Seq, a proteomic methodology in which serum antibodies are purified by affinity chromatography against an immobilized antigen—in this case, immobilized GII.4c VLP—then proteolytically digested into peptides and analyzed by liquid chromatography-tandem mass spectrometry (LC-MS/MS). Peptide spectral matches were obtained using a custom database of heavy-chain-variable (VH) genes encoded by peripheral B cells from the respective donor. To construct the VH database, peripheral blood mononuclear cells (PBMCs) collected at each time point were split into two aliquots: one aliquot was sequenced using high-throughput single-cell flow-focusing technology (DeKosky et al., 2015; McDaniel et al., 2016) to determine the native VH/VL (heavy-chain-variable/light-chain-variable) paired repertoire, and the second aliquot was processed to determine the VH repertoire. Relative quantitation of serum anti-GII.4c antibodies was determined from the LC peak intensity of high-confidence peptide-spectrum matches (PSMs) derived from peptides of the third complementarity determining region of the heavy chain (CDR-H3). Antibodies belonging to a CDR-H3 clonotype likely recognize the same epitope, given the dominant role of CDR-H3 in antigen recognition. As we established earlier, the Ig-Seq workflow identifies the majority (>70%) of the most abundant antigen-specific antibodies in the serum, and quantitation calibrations using isobaric peptide spike-ins show that peak intensities correlate well with absolute peptide concentrations (Lavinder et al., 2014). The antibody repertoire composition and relative quantities at the clonotypic level are plotted as a histogram with each bar representing an individual clonotype and the horizontal axis showing its relative abundance (Figure 1B). All three donors had a titer to GII.4c at day 0, presumably a consequence of prior exposure to GII.4 infection and consistent with earlier data indicating that the vast majority of asymptomatic adults have antibodies to HuNoV (Carmona-Vicente et al., 2015; Nurminen et al., 2011). As has been the case with the serological repertoire to other antigens reported so far (e.g., influenza hemagglutinin [Lee et al., 2016], tetanus toxoid [Lavinder et al., 2014], or desmoglin in pemphigus patients [Chen et al., 2017]), the repertoire to GII.4c was found to be oligoclonal and highly polarized, in that <3 antibodies dominated the response, contributing more than 50% of the antigen-specific titer (Figure 1B). In particular, at day 0, the dominant CDR-H3 clonotype in each repertoire accounted for between 58% and 86% of the total CDR-H3 peptide intensity, suggesting that the long-term serological memory to GII.4 is dominated by a single antibody. Other examples of highly skewed serological repertoire polarization with one clonotype accounting for >60% of the response have been reported previously (Lee et al., 2016).

Antibody clonotypes present in both the pre-vaccination repertoire (day 0) and the post-vaccination repertoire were defined as "pre-existing," whereas clonotypes observed exclusively in the post-vaccination repertoire were defined as "emergent." The term "emergent antibodies" refers only to the

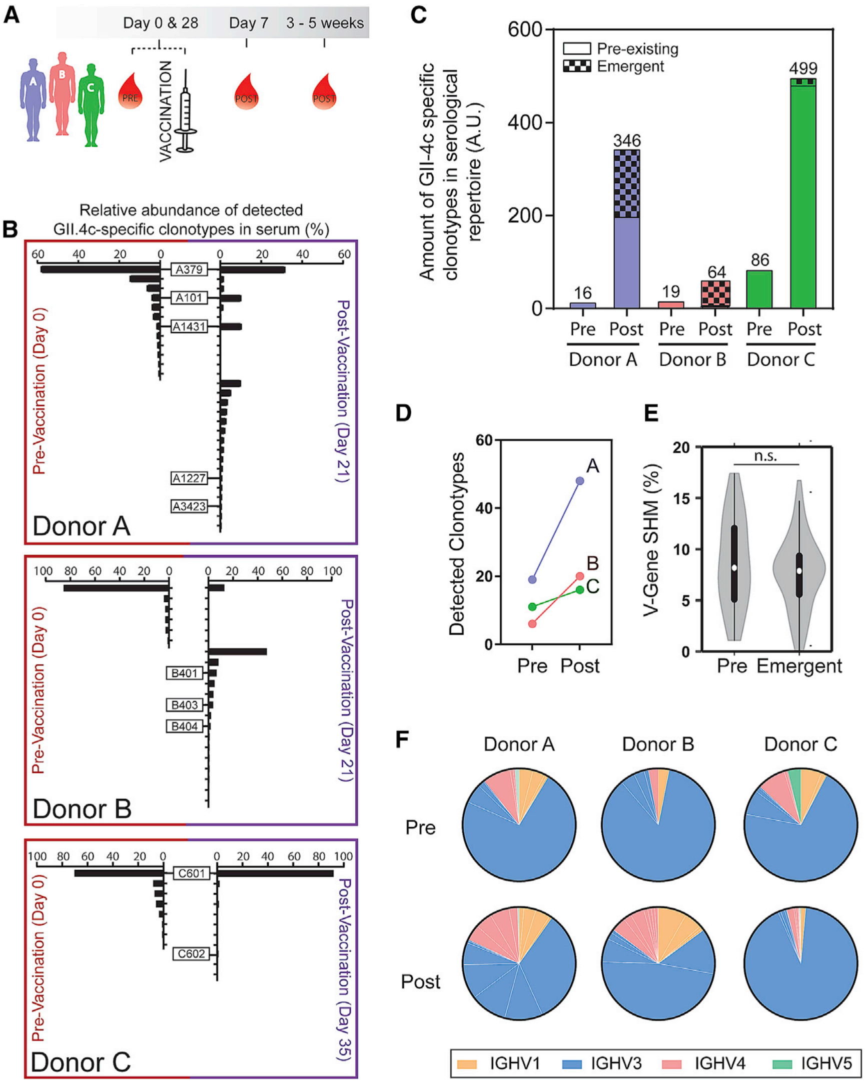

Figure 1. The Anti-NoV GII.4c IgG Serum Antibody Repertoire following Vaccination Is Polarized and Shaped by Previous HuNoV Infection

(A) Donors (n = 3) were immunized with experimental HuNoV vaccine and serum and PBMCs were collected at the times indicated.

(B) Relative abundance of GII.4c-specific IgG clonotypes pre- and post-vaccination. Each bar represents a CDR-H3 antibody clonotype observed at ≳0.5% of the GII.4c antigen-specific antibody repertoire.

(C) Amount of anti-GII.4c serum antibodies (i.e., the product of anti-GII.4c IgG ELISA titer × relative antibody abundance as determined by LS-MS/MS; see STAR Methods) pre- and post-vaccination; ELISA titer shown over each bar.

(D) Number of detected antibody clonotypes before and after vaccination.

(E) Anti-GII.4c antibody V-gene somatic hypermutation rates for pre-existing and emergent antibodies; n.s. represents not statistically significant (two-tailed t test; $p > 0.3$).

(F) V-gene family usage in the serological repertoire.

Related to Table S1.

fact that they become detectable in the serum post vaccination and should not be interpreted in terms of B cell ontogeny (i.e., whether they arise from stimulated naive cells or memory B cells). Although vaccination increased the diversity of the serological repertoire in all donors, both the number and abundance of vaccine-elicited clonotypes were substantially lower in donor C, who had a 4- to 5-fold higher titer to GII.4c before vaccination (day 0) compared to the other two donors (Figures 1B–1D). In this donor, the increase in the GII.4c titer 4 weeks post vaccination was overwhelmingly due to the boosting of the dominant antibody clonotype C601, which accounted for >90% of the serum response and increased in absolute abundance by more than 7-fold relative to the level observed at day 0. The low level of emergent antibodies in donor C, who had the highest pre-vaccination titer, is consistent with the strong inverse correlation between day 0 serum titer and the number of emergent antibody clonotypes in the serological memory after immunization with the seasonal influenza vaccine (Lee et al., 2016).

Pre-existing and emergent antibodies displayed similar somatic hypermutation profiles (Figure 1E). Although emergent antibodies (7.1 ± 0.5% mean rate of somatic hypermutation) were slightly less mutated than the pre-existing antibodies (8.2 ± 1.1%), this difference did not reach statistical significance. The anti-GII.4c serological repertoire was predominantly comprised of antibodies using the immunoglobulin heavy variable 3 (IGHV3) family (~80%; Figure 1F) compared to ~30% in naive or mature B cells (DeKosky et al., 2016; Mroczek et al., 2014). These findings suggest that the serological IgG response to human norovirus vaccination is largely dominated by the boosting of a limited number of pre-existing antibody clonotypes.

Recombinant HuNoV Serum Antibodies Demonstrate Neutralization against GII.4 and Breadth of Binding across Strains

We expressed and characterized a set of 10 representative serum antibodies for which both VH and VL sequences were detected by paired VH/VL sequencing. These included the top most abundant clonotypes in donors A and C (A379 and C601), two strongly boosted pre-existing clones (A101 and A1431, boosted by 12× and 126×, respectively, in terms of abundance in the post-vaccination serum relative to day 0), and six emergent antibodies (Figure 1B). Binding and blockade activity were tested using an antigenically diverse panel of norovirus genogroup I and genogroup II VLPs, including a time-ordered panel of pandemic GII.4 VLPs representing historical variant strains, including US95_96 (GII.4 1997), Farmington Hills (GII.4 2002), and Den Haag (GII.4 2006b), as well as prospective strains that emerged following vaccine design (2006) and the

clinical trial (2010), such as GII.Pe-GII.4 Sydney (GII.4 2012) and GII.P16-GII.4 Sydney (GII.4 2015) (Lindesmith et al., 2018a). As expected, all antibodies bound to the vaccine strain VLP (Figure 2A), with half-maximal effective concentration (EC_{50}) values between 8 and 717 ng/mL. The potential for neutralization by inhibition of cellular ligand binding was evaluated by determining blockade of VLP-ligand binding (blockade assay) (Figure 2B). The 10 serum antibodies could be categorized into three classes. The first class consisted of 6 antibodies that displayed broad binding but no blocking activity toward multiple GI and GII strains; three of these, namely A1227, B401, and B404, showed binding to all strains tested, including a variety of GI and GII genotypes such as GI.I, GII.2, and GII.17. The VP1 protein among these strains shows only ~50% amino acid identity (Zheng et al., 2006). The second class consisted of antibodies B403, C601, and C602, which had strong blockade activity towards the vaccine strain and the earlier pandemic GII.4 1997 strain but not to GII.4 strains that emerged post vaccination. Finally, the third class of serum antibodies had a single member, A1431, which had strong blockade activity to all known historical GII.4 strains from 1987 onwards. A1431 had very extensive breadth and potency not only towards all historical strains available but also towards prospective GII.4 strains that appeared after the vaccine trial, including GII.4 2012 and 2015, neither of which had circulated in the general population at the time of sample collection (Figure 2B). Furthermore, A1431 potently neutralized GII.P16-GII.4 Sydney virus, a GII.4 strain that emerged 5 years post sample collection, in a human intestinal enteroid culture system (Costantini et al., 2018; Ettayebi et al., 2016) (IC_{50} 5.5 ng/mL), supporting the correlation between blockade of ligand-binding potency and antibody-mediated neutralization (Figures 2C and S1) (Alvarado et al., 2018; Ettayebi et al., 2016).

We sought to define biochemically the epitopes recognized by the antibodies with blockade activity to historical strains, namely B403, C601, and C602; as discussed below, the epitope of A1431 that has broad blockade breadth was determined by crystallography. We first evaluated antibody mediated blockade against a panel of VLPs with epitopes A, D, and E exchanged from GII.4 2012 (non-binding VLP) into GII.4 1987 (binding VLP). Exchange of epitope A residues ablated blockade potency of B403 and diminished potency of C601 by 13.5-fold but had little effect on C602 blockade (1.8-fold decrease). Exchange of epitope D or E residues had little impact on blockade of any of the three antibodies, suggesting that the major binding determinants for B403 and C601 likely reside within epitope A (Figures 3A–3C). VLPs displaying an acidic residue at 298, Val at position 356, and His at 357 had lower EC_{50} titers than VLPs without these residues for all three antibodies (Figures 3B and 3C). Exchange of V298E/A356V/E357H into the null backbone VLP 581 (Lindesmith et al., 2018b) (VLP 581.A4A) gained binding of B403 and C601 and improved binding of C602 by 11.6-fold compared to

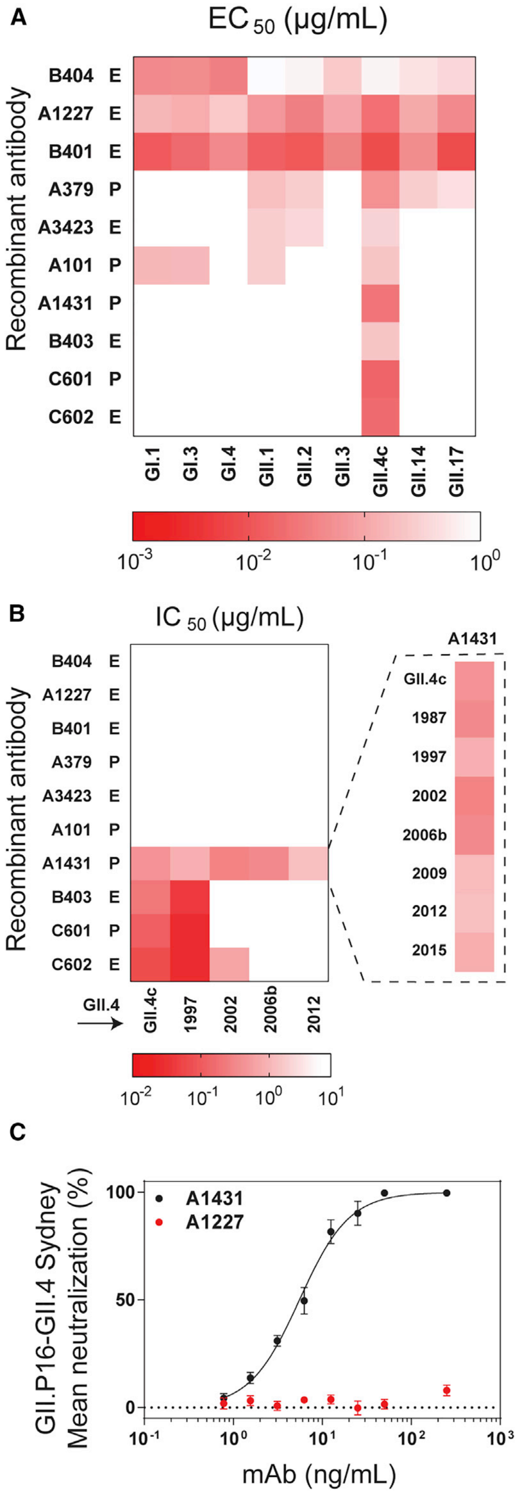

Figure 2. HuNoV Recombinant Serum Antibodies Recognize Conserved Binding, Ligand-Blocking, and GII.4 Neutralizing Epitopes

Sigmoidal dose response curves were fit to the mean percent control binding for determination of (A) half-maximum binding (EC_{50}) or (B) half-maximum blocking of HBGA binding (half-maximum inhibitory concentration, IC_{50}) of pre-existing (P) and emergent (E) antibodies. Titers >2 µg/mL were scored as negative (white box). The HBGA blocking assay was used to characterize A1431 against a panel of pandemic norovirus GII.4 strains. Antibodies were tested in duplicate in a minimum of two independent experiments. (C) Sigmoidal dose response curves were fit to the mean percent viral genomic copies control for determination of IC_{50} in a neutralization assay. The neutralization assay was used to characterize A1431 and A1227 against the GII.P16-GII 4 Sydney strain. Error bars represent mean ± SEM from four independent experiments with 2 technical replicates for each dilution. Related to Figure S1.

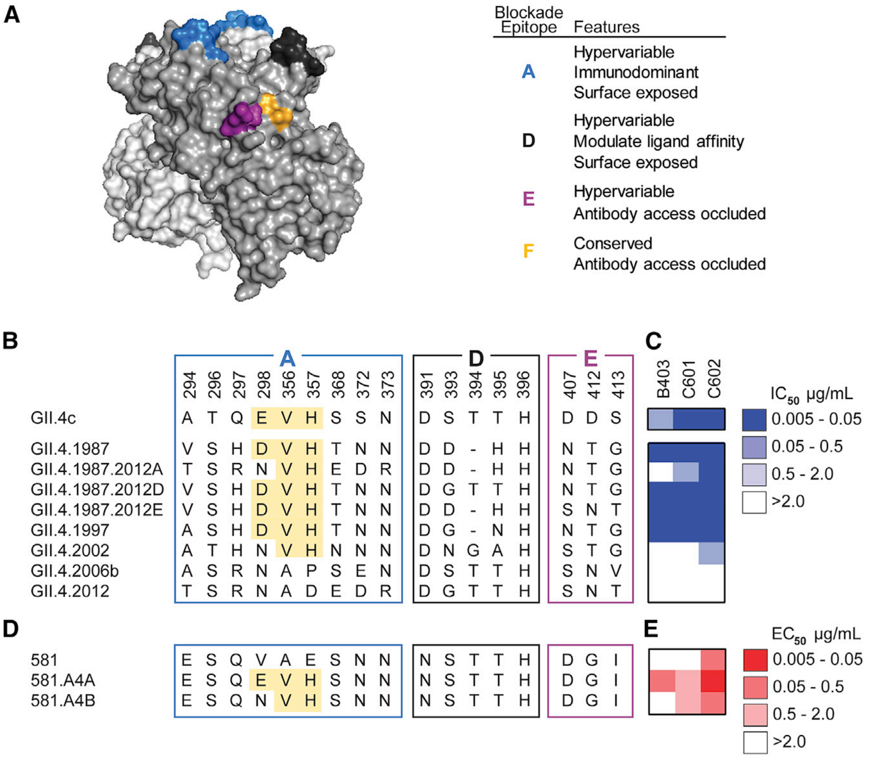

Figure 3. Serum Antibodies that Block Historical GII.4 Strains Recognize Overlapping Epitopes within Epitope A

(A) Homology model based on PDB: 3SLD (Shanker et al., 2011) of the GII.4 2006a P domain dimer of the HuNoV major capsid protein, VP1 with known P domain blockade antibody epitopes A (blue), D (dark gray), E (purple), and F (orange) highlighted.

(B) Amino acid sequences of pandemic strains and select mutants of GII.4 1987.

(C) Blockade potency binned by IC_{50}.

(D) Select mutants of GII.4 2006a.581 VP1. Highlighted areas indicate sequences important for antibody binding.

(E) Antibody binding binned by EC_{50} values. Antibodies were tested in duplicate in a minimum of two independent experiments.

the 581 VLP (Figures 3D and 3E). Substitution of V298N/A356V/E357H (VLP 581.A4B) did not gain binding for B403, yielded similar binding to that to 581.A4A for C601, and decreased C602 binding by 7.6-fold compared to 591.A4A. These data confirm E or D at position 298 as an anchor residue for B403. For antibody C601, binding was heavily influenced by V356/H357, residues that are predicted to contribute to epitope A but have not been previously verified. Further, our analysis also revealed that C602 recognizes a previously undefined blockade epitope. Together, these data categorize antibodies boosted by HuNoV VLP vaccination into three classes: (1) genogroup I and genogroup II broadly binding, non-neutralizing antibodies; (2) ancestral GII.4 binding and ligand-blocking antibodies; and (3) GII.4 broadly binding and neutralizing antibodies. Genetic mapping indicated that antibodies displaying a limited breadth of GII.4 ligand-blockade potency (class 2 above) recognized the immunodominant epitope A antigenic site (Mallory et al., 2019) and accounted for previously observed back-boost antibody responses in HuNoV vaccinated donors (Lindesmith et al., 2015). These data demonstrate that HuNoV vaccination can elicit serum antibodies capable of neutralizing GII.4 pandemic strains.

Structural Studies Define a Highly Conserved Non-Blocking Epitope and a Conserved GII.4 Neutralizing Epitope within the Capsid P Domain

To gain insight into the molecular mechanisms underlying cross-reactive, non-blockade activity, we selected the pan-reactive antibody A1227 for crystallographic analysis. We purified the antigen-binding fragment (Fab) of A1227 in complex with soluble GII.4 P domains from 2002, 2006, 2009, or 2012 pandemic strains and screened for crystallization. Complexes with the GII.4.2002 P domain (P2002) yielded diffracting crystals suitable for structural analysis.

Single crystals of the complex between A1227 and P2002 diffracted to 2.6 Å, and the structure was solved by molecular replacement using the apo P2002 structure (PDB: 4OOV) (Singh et al., 2015) (Table S2). A1227 Fab bound to each of the conserved P1 subdomains of the P2002 dimer (Figure 4A). Each Fab bound at the interface between monomers in the P2002 dimer, with the heavy chain recognizing both protomers and the light chain recognizing only a single protomer (Figure 4B). A1227 recognized a region of P2002 which was mostly conserved across genogroups GI and GII (Figures 4C and S2), thus explaining the broad cross-reactivity of antibody A1227. Overall, each Fab buried ~950 $Å^2$ by establishing polar contacts and favorable van der Waals interactions with P2002 (Figures 4D and S2). There were no substantial conformational changes upon A1227 binding to P2002, with a root-mean-square deviation (RMSD) of 0.6 Å between apo-P2002 and A1227-P2002 complex structures.

To map the A1227 epitope within the context of the VLP, the crystal structure of the A1227-P2002 complex was superimposed onto the GI.1 capsid structure (PDB: 1IHM) (Prasad et al., 1999). The GI.1 capsid structure was chosen as a template for modeling due to similarities between GI.1 and GII.4 capsid structures indicated by prior electron microscopy (EM) studies (Chen et al., 2004). Modeling revealed that the conserved region recognized by A1227 was located at an occluded site on the norovirus capsid, at the interface between the P domain and shell domain. Overlay of Fab A1227 onto the GI.1 capsid structure (Figures 4E and 4F) indicated that other P domains on the assembled norovirus capsid would clash with both heavy and light chains of the Fab, with only minor clashes with the shell domains (Figure 4G), suggesting that structural rearrangements of the P domain could either expose the A1227 binding site or allow binding at a neighboring P domain. Its lack of blockade or neutralizing activity likely results from antibody A1227 only recognizing defects or maturation intermediates (Pogan et al., 2018).

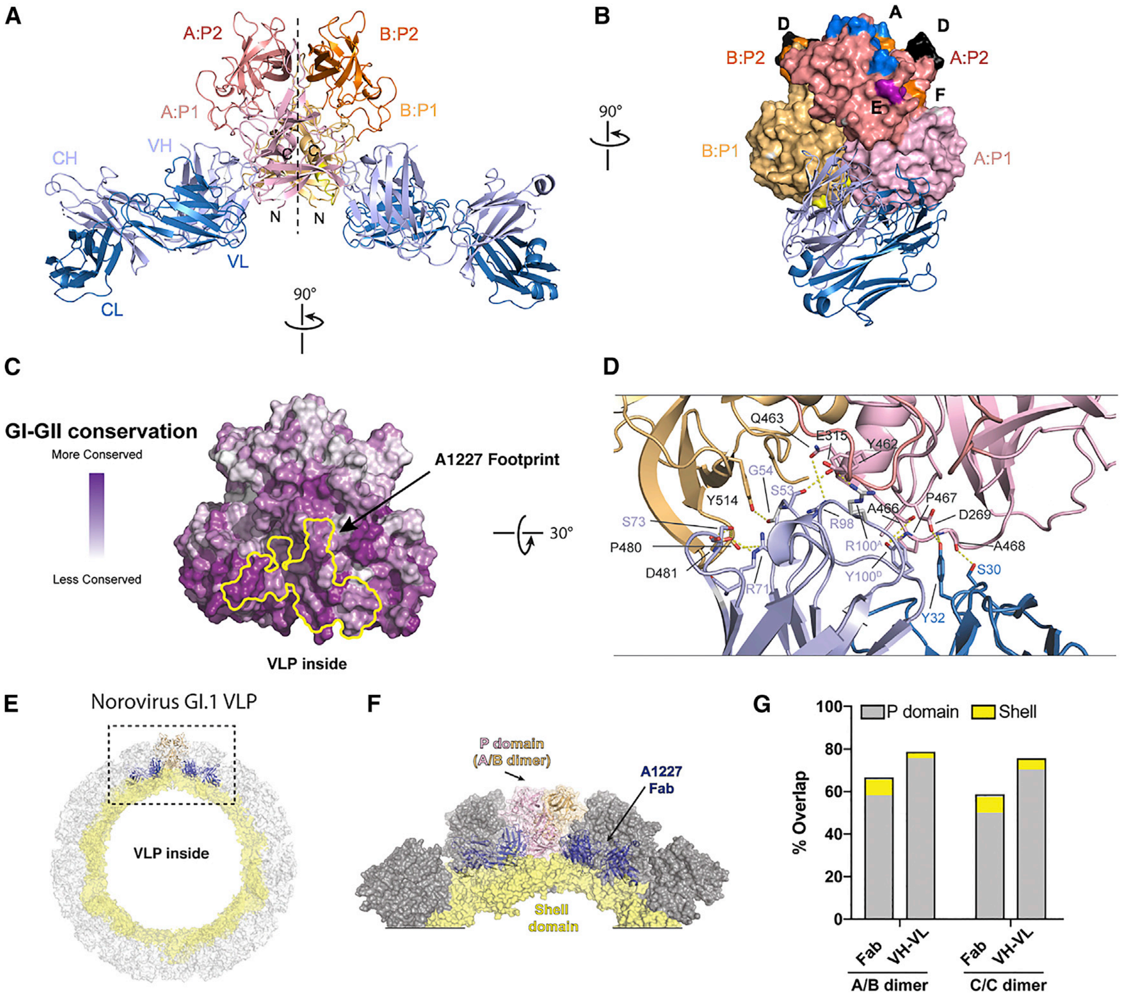

Figure 4. The Broadly Binding Non-Blockade Antibody A1227 Binds to a Highly Conserved Epitope within the P Domain

(A) Crystal structure of A1227 Fab bound to GII.4.2002 P domain dimer shown in cartoon representation, with P domain monomers colored pink and orange and A1227 heavy and light chains in gray and blue, respectively.

(B) The A1227 antibody binds near the P1 dimeric interface (P domain dimer in surface representation and A1227 Fab in cartoon).

(C) A1227 Fab footprint is outlined in yellow on the P domain surface, mapped by GI-GII sequence conservation, with degree of conservation colored from variable (white) to conserved (purple).

(D) Detailed view of A1227 Fab-P domain binding interface, with interacting residues depicted in stick and hydrogen bonds shown in yellow dotted lines.

(E) Modelling of the GII.4.2002 P domain-A1227 Fab crystal structure onto the GI.1 capsid structure (PDB: 1IHM) reveals an antibody binding site that is occluded on the assembled VLP.

(F) Close-up view from (E) showing the occlusion of A1227 Fab within the GI.1 capsid.

(G) Quantification of volume overlap between A1227 Fab (or VH/VL) and GI.1 capsid proteins. Over 60% of the volume of A1227 Fab overlaps with neighboring P domains when modelled onto either A/B or C/C dimers. Only small overlaps are due to clashing with shell domains. Overlaps from P domain and shell domain are colored in gray and yellow, respectively. Volumes were calculated using Voss-Void-Voxelator program (Voss and Gerstein, 2010).

Related to Table S2 and Figure S2.

To provide a structural basis for recognition by the GII.4 broad blockade antibody A1431, we solved the crystal structure of A1431 Fab in complex with the GII.4.2002 P domain at 3.1 Å resolution (Table S2). The asymmetric unit contained two P-domain-A1431 Fab complexes, with each dimer generated by crystallographic 2-fold symmetry. Both complexes were nearly identical, with an RMSD of 0.3 Å. Electron density for both P2002 and A1431 variable domains was well defined, although regions of A1431 constant domains were not well ordered.

Antibody A1431 bound each P domain monomer at a cleft between the P1 and P2 subdomains (Figure 5A). Similar to A1227, A1431 binding did not induce substantial conformational changes in the P domain, with an overall RMSD of 0.4 Å between the complex and apo (PDB: 4OOV) structures. Antibody A1431 recognized an epitope located near previously mapped blockade epitopes D and F and distinct from the site of HBGA binding (Figure 5B). Both A1431 heavy and light chains contributed to the binding interface with a total buried surface area of ~985 $Å^2$ on the P domain. The CDR-H3 loop of A1431 was

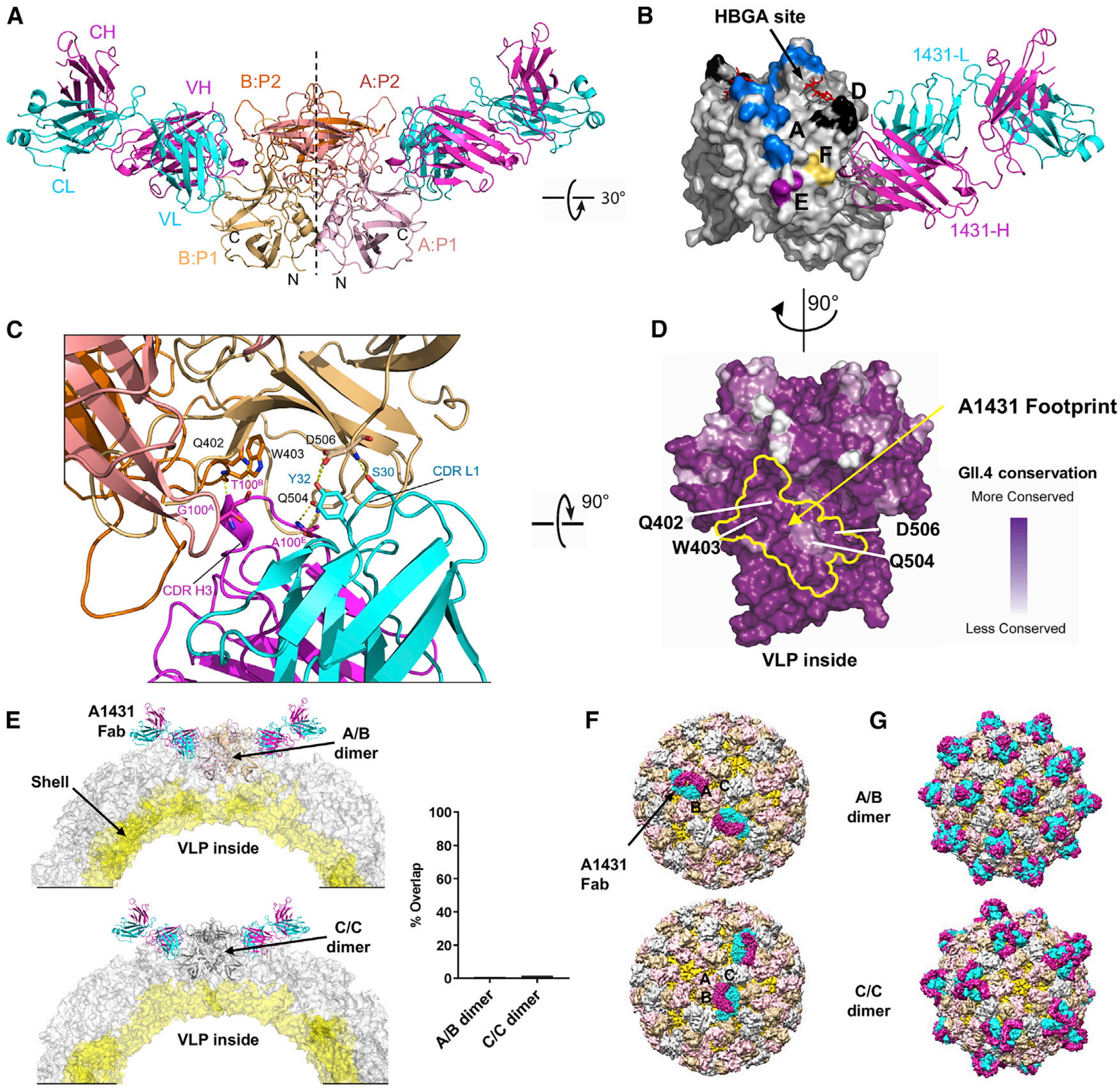

Figure 5. Broad GII.4-Blockade Antibody A1431 Recognizes an Accessible Semi-Conserved Site of Vulnerability

(A) Overall structure of A1431 Fab bound to GII.4.2002 P domain dimer is shown in ribbon representation, with P domain monomers in pink and orange and A1431 heavy and light chains in magenta and cyan, respectively.

(B) Mapped blockade epitopes are highlighted on a surface representation of GII.4.2002 P domain dimer (colored as in Figure 3A) with the A1431 Fab shown in cartoon. The crystal structure of GII.4 (Farmington Hills, 2004) P domain complexed to HBGA type B ligand (PDB: 4X05) was superimposed onto the GII.4.2002 P domain + A1431 complex to show location of HBGA binding site.

(C) Details of A1431 Fab interactions with P domain residues are highlighted with interacting residues depicted in stick and hydrogen bonds shown in yellow dotted lines.

(D) A1431 Fab footprint is outlined in yellow on the P domain dimer surface, mapped by GII.4 sequence conservation, with degree of conservation colored from variable (white) to conserved (purple).

(E) The GII.4.2002 P domain-A1431 Fab crystal structure is modeled onto the GI.1 capsid structure (1IHM; Prasad et al., 1999), with a cross-section showing the P domain fit onto the A/B dimer (top) or the C/C dimer (bottom). Overlap between A1431 Fab and P domain, as modeled onto either GI.1 capsid A/B or C/C dimers, is minimal and shown on right, as in Figure 4G.

(F) Modeling of A1431 Fab to the A/B dimer (top) or C/C dimer (bottom) on the GI.1 capsid structure, with A/B dimers colored pink and tan, C/C dimers colored in gray and shell domain shown in yellow.

(G) GI.1 capsid models, colored as in (F), showing occupancy of A1431 binding sites on all A/B dimers (top) or all C/C dimers (bottom).

Related to Table S2 and Figure S3.

inserted into a cleft between the P1:P2 subdomains and was held in place by hydrogen bonding between main-chain atoms of the CDR-H3 and P domain residues Gln402, Trp403, and Gln504 (Figure 5C). Ser30 and Tyr32 from the CDR-L1 loop provide additional stabilization via hydrogen bonds to Asp506 on the P1 subdomain. The recognized surface, including P domain residues Gln402, Trp403, Gln504, and Asp506, was nearly completely conserved in GII.4 (Figure 5D) but only semi-conserved between GI and GII genogroups, with Asp506 changed to Pro506 in most other GI and GII strains (Figure S3),

providing the structural basis for the broad recognition of A1431 of GII.4 viruses but not of other norovirus genotypes.

To gain insight into the molecular mechanism of blockade activity by antibody A1431, we superimposed the crystal structure of the P2002-A1431 Fab complex onto the GI.1 capsid structure (Figure 5E). On the assembled VLP, the P domain dimer exists in two distinct structural environments, defined by A/B and C/C dimers (Prasad et al., 1999). A1431 Fab binding to the A/B dimer showed no overlap between Fab and either P or S domains, whereas binding to the C/C dimer showed minor overlap (Figures 5E and 5F). Binding of the A1431 Fab to all equivalent sites on the VLP showed recognition at the A/B dimer to cover most of the VLP surface, whereas recognition of the C/C dimer left the 5-fold vertices potentially capable of attaching to HBGAs at the cell surface (Figure 5G). In both cases, binding at all sites would lead to substantial antibody-antibody overlap (Figures 5F and 5G), suggesting that A1431 exerts its blockade and neutralizing activity by binding to only a fraction of potential binding sites on the VLP. Overall, in contrast to the A1227 binding site, modeling suggested the A1431 blockade epitope to be mostly accessible on the capsid surface. Notably, the A1431 epitope did not overlap with identified sites of HBGA recognition, and therefore, the observed blockade functionality likely stems either from antibody A1431 sterically inhibiting the approach of the VLP to the >500 kDa multivalent HBGAs found on MUC2 in PGM and in human gastric and duodenal tissues (Park et al., 2015) or from subtle allosteric effects, as reported for another antibody that docks near to the 1431 binding site (Lindesmith et al., 2014, 2018b), as defined by epitope mapping. High-resolution structures of broadly reactive and/or neutralizing epitopes expand our understanding of the HuNoV antigenic landscape and narrow the search for optimal vaccine candidates. For instance, whereas antibodies targeting hypervariable P2 epitopes (i.e., epitope A) have limited cross-strain ligand-blocking potency and antibodies recognizing highly conserved epitopes near the P1-shell-domain interface are highly cross-reactive but have no blocking potency, the antibodies targeting genotype-conserved epitopes at the P1-P2 interface display broad ligand-binding blocking potency. It should be noted that each of these antibody classes may contribute to vaccine-induced protection, and further studies and *in vivo* models are required to fully understand the interplay between the antibody targets and the physiological response.

DISCUSSION

Vaccine-induced immunity relies on the development and maintenance of long-term protective antibody titers or serological memory. The best studied correlate for protective immunity after HuNoV infection is the development of robust blockade antibody responses (Lindesmith et al., 2015; Malm et al., 2014; Reeck et al., 2010). Immune responses to rapidly evolving viral infections are complex and heavily impacted by pre-exposure histories that shape the antibody response to newly evolved strains (Belongia et al., 2017; Gostic et al., 2016; Lessler et al., 2012; Lindesmith et al., 2017). These studies demonstrate that bivalent HuNoV vaccines can elicit GII.4 neutralizing antibody responses in humans, while also structurally defining the highly conserved GII.4 (1987–2015) epitope recognized by a neutralizing antibody.

Here, we demonstrate that the serological repertoire to HuNoV is oligoclonal and dominated by IGHV3 family antibodies in three pre-immune individuals who received the vaccine. A large fraction (6/10) of the serum antibodies that we biochemically characterized in detail displayed significant binding breadth, with 4/6 recognizing both GI and GII genotype VLPs. Although these high-binding-breadth antibodies did not have blockade activity, they may play a role in protection against HuNoV infection via Fc-mediated mechanisms. Non-neutralizing antibodies that protect against infection in animal models are observed at significant levels in circulation after influenza vaccination (Lee et al., 2016), and recent studies report the isolation of such antibodies for other viruses as well (Bootz et al., 2017; Henry Dunand et al., 2016). Current technologies do not allow direct evaluation of HuNoV Fc-mediated protection.

In donor A, we showed that the most dramatically back-boosted blockade antibody following vaccination is capable of blocking VLP-HBGA binding of all examined GII.4 strains circulating between 1987 and 2015 and is potently neutralizing in the human intestinal enteroid culture system. This antibody was present in appreciable amounts in the serum prior to vaccination, suggesting that it must have been elicited by an earlier HuNoV infection. Although lack of successful propagation of historic GII.4 strains limits our ability to test the breadth of GII.4 neutralization, all tested blockade monoclonal antibodies have also been neutralizing antibodies (Alvarado et al., 2018), indicating that A1431 is also likely cross-GII.4 neutralizing. These findings reveal that HuNoV infection can induce broad blockade and neutralizing antibodies and that these antibodies can be boosted by vaccination (Lindesmith et al., 2012a, 2015).

A1431 is a broad blockade neutralizing human antibody for which the binding site has now been structurally defined. The structures of antibodies A1431 and A1227 in complex with the P domain described here, as well as structures of the GI.1-specific, HBGA-blocking 5I2 IgA (Shanker et al., 2016) and of the broadly GII-reactive 5B18 IgG (Hansman et al., 2012) described previously, support multiple mechanisms of antibody-mediated recognition and potentially protection from norovirus infection (Koromyslova et al., 2018). The most abundant ligand-blocking antibodies recognize hypervariable surface-exposed epitopes that are frequently mutated as a result of antigenic drift (Debbink et al., 2012a; Lindesmith et al., 2013, 2015). Antibody binding of these epitopes blocks the HBGA binding pocket (Shanker et al., 2016). In contrast, A1431 recognizes conserved residues distal to the HBGA binding pocket but accessible on the assembled VLP. Blocking of ligand binding and neutralization likely results not from direct interference at the ligand-binding site but rather from either prevention of attachment to the ligand-displaying surface (i.e., steric effects) or modulation of capsid dynamics (Lindesmith et al., 2014, 2018b). We note that the unprecedented blockade breadth of A1431 may be useful for therapeutic purposes, as currently there are no treatment options for chronic infection or life-threatening HuNoV infection (Ahmed et al., 2012; Cardemil et al., 2017; Petrignani et al., 2018). The co-crystal structure of A1431 with the P domain potentially provides avenues by which this antibody may be further improved for breadth and potency (Diskin et al., 2011; Kwon et al., 2018). Antibodies like A1227 with broad cross-genogroup binding have not yet been shown to neutralize or block VLP binding to

HBGAs. These antibodies likely bind to occluded epitopes available for antibody access during phases of particle assembly and/or disassembly, intermediates, or rearrangements. Although mechanisms of protection driven by this class of antibodies are undefined, short-term boosting of cross-blockade antibodies after vaccination (Lindesmith et al., 2015) and mouse norovirus (MNV) monoclonal antibodies with cross-serotype neutralization (Kolawole et al., 2017) support the potential for HuNoV cross-genotype neutralization.

These data provide structural and mechanistic evidence supporting that the GII.4 component of the Takeda multivalent norovirus VLP vaccine recalls a memory antibody response that persists for at least six months, indicating that, similar to other rapidly evolving RNA viruses, early-life norovirus strain exposure shapes antibody responses to subsequent norovirus strains (Lindesmith et al., 2015, 2017). As with influenza, exposure history and genetic background are expected to impact outcomes differently by age groups, potentially impacting vaccine efficacy (Gostic et al., 2016; Lessler et al., 2012). Future studies are needed to determine additional mechanisms of the antibody boost to prospective GII.4 strains, as well as the antibody response in key vaccine target populations, including the aged, with a life-time of norovirus exposure but waning immune responsiveness, and the very young, with no pre-exposure history and possibly with maternal antibodies. These data are critical for understanding vaccine performance and population-based immunity against future pandemic isolates. Cross-blocking antibodies like A1431 provide one mechanism of breadth, and serological repertoire analyses offer robust strategies to dissect the molecular basis for this and other responses in human populations. Coupled with blockade, neutralization potency in the enteroid culture system (Costantini et al., 2018; Ettayebi et al., 2016), and structural determination of antibody binding sites, recombinant serum antibodies can provide invaluable information for immunogen design and vaccination strategies that phenocopy infection and provide broad, long-term protection to HuNoV pandemics.

STAR★METHODS

Detailed methods are provided in the online version of this paper and include the following:

- KEY RESOURCES TABLE
- CONTACT FOR REAGENT AND RESOURCE SHARING
- EXPERIMENTAL MODEL AND SUBJECT DETAILS
  - Humans Subjects
  - Cell lines
  - Microbes
- METHOD DETAILS
  - Ten percent stool filtrate
  - Human intestinal enteroid culture (HIE)
  - High-throughput sequencing of BCR transcripts
  - VH Sequencing
  - VH/VL Sequencing
  - Isolation of HuNoV specific antibodies
  - Sample preparation and LC-MS/MS analysis
  - Antibody expression and purification
  - Production of Virus Like Particles
  - Antibody functional assays
  - P domain expression and purification
  - Crystallization of Fab complex with P2002
  - Structure determination and analysis
  - Neutralization Assay
- QUANTIFICATION AND STATISTICAL ANALYSIS
- DATA AND SOFTWARE AVAILABILITY

SUPPLEMENTAL INFORMATION

Supplemental Information can be found online at https://doi.org/10.1016/j.immuni.2019.05.007.

ACKNOWLEDGMENTS

The authors would like to thank Takeda Vaccines for provision of human samples; the Genome Sequencing and Analysis Facility at the University of Texas at Austin for performing Illumina next-generation sequencing; the Center for Biomedical Research Support Proteomics Facility at the University of Texas at Austin for providing mass spectrometry analysis; and Victoria Madden of Microscopy Services Laboratory, Department of Pathology and Laboratory Medicine, University of North Carolina–Chapel Hill for expert technical support. Support for this work was provided by the Intramural Research Program of" the Vaccine Research Center, NIAID, NIH, and the intramural food safety program at CDC and by grants from the NIH (R56AI106006, G.G. and R.S.B. and U19 AI109761 CETR, R.S.B.), Defense Threat Reduction Agency (DTRA) (contract HDTRA1-12-C-0105, G.G.), and the Wellcome Trust [203268/Z/16/Z] (R.S.B.). Use of sector 22 (Southeast Region Collaborative Access team) at the Advanced Photon Source was supported by the U.S. Department of Energy, Basic Energy Sciences, Office of Science, under contract number W-31-109-Eng-38. The findings and conclusions in this article are those of the authors and do not necessarily represent the official position of the Centers for Disease Control and Prevention. The funders had no role in study design, data collection and interpretation, or the decision to submit the work for publication.

AUTHOR CONTRIBUTIONS

Conceptualization: R.S.B., G.G., L.C.L., J.R.M., A.C., R.V., S.A.K., and P.D.K. Investigation and Formal Analysis: L.C.L., J.R.M., A.C., R.V., S.A.K., V.C., P.D.J.B., M.L.M., W.N.V., D.R.B., J.J.B., G.C.I., and J.V. Writing, All Drafts: L.C.L., J.R.M., A.C., R.V., P.D.K., G.G., and R.S.B. with input from all authors. Funding Acquisition and Supervision: R.S.B., G.G., and P.D.K.

DECLARATION OF INTERESTS

J.R.M., S.A.K., A.C., R.V., V.C., P.D.B.J., M.L.M., W.N.V., D.R.B., J.J.B., G.C.I., J.V., P.D.K., and G.G. declare no competing interests. L.C.L. and R.S.B. have consulted and collaborated with Takeda on norovirus vaccines. They are also inventors on patents filed on issues relevant to noroviruses. G.G. is an inventor on patents related to the analysis of the serological antibody repertoire.

Received: January 3, 2019
Revised: March 15, 2019
Accepted: May 15, 2019
Published: June 13, 2019

SUPPORTING CITATIONS

The following references appear in the Supplemental Information: Word et al. (1999).

REFERENCES

Adams, P.D., Gopal, K., Grosse-Kunstleve, R.W., Hung, L.W., Ioerger, T.R., McCoy, A.J., Moriarty, N.W., Pai, R.K., Read, R.J., Romo, T.D., et al. (2004).

Recent developments in the PHENIX software for automated crystallographic structure determination. J. Synchrotron Radiat. *11*, 53–55.

Agnihothram, S., Menachery, V.D., Yount, B.L., Jr., Lindesmith, L.C., Scobey, T., Whitmore, A., Schäfer, A., Heise, M.T., and Baric, R.S. (2018). Development of a Broadly Accessible Venezuelan Equine Encephalitis Virus Replicon Particle Vaccine Platform. J. Virol. *92*, e00027–e00018.

Ahmed, S.F., Klena, J.D., Mostafa, M., Dogantemur, J., Middleton, T., Hanson, J., and Sebeny, P.J. (2012). Viral gastroenteritis associated with genogroup II norovirus among U.S. military personnel in Turkey, 2009. PLoS ONE *7*, e35791.

Alvarado, G., Ettayebi, K., Atmar, R.L., Bombardi, R.G., Kose, N., Estes, M.K., and Crowe, J.E., Jr. (2018). Human Monoclonal Antibodies That Neutralize Pandemic GII.4 Noroviruses. Gastroenterology *155*, 1898–1907.

Atmar, R.L., Bernstein, D.I., Harro, C.D., Al-Ibrahim, M.S., Chen, W.H., Ferreira, J., Estes, M.K., Graham, D.Y., Opekun, A.R., Richardson, C., and Mendelman, P.M. (2011). Norovirus vaccine against experimental human Norwalk Virus illness. N. Engl. J. Med. *365*, 2178–2187.

Atmar, R.L., Ramani, S., and Estes, M.K. (2018). Human noroviruses: recent advances in a 50-year history. Curr. Opin. Infect. Dis. *31*, 422–432.

Baric, R.S., Yount, B., Lindesmith, L., Harrington, P.R., Greene, S.R., Tseng, F.C., Davis, N., Johnston, R.E., Klapper, D.G., and Moe, C.L. (2002). Expression and self-assembly of norwalk virus capsid protein from venezuelan equine encephalitis virus replicons. J. Virol. *76*, 3023–3030.

Belongia, E.A., Skowronski, D.M., McLean, H.Q., Chambers, C., Sundaram, M.E., and De Serres, G. (2017). Repeated annual influenza vaccination and vaccine effectiveness: review of evidence. Expert Rev. Vaccines *16*, 1–14.

Bernstein, D.I., Atmar, R.L., Lyon, G.M., Treanor, J.J., Chen, W.H., Jiang, X., Vinjé, J., Gregoricus, N., Frenck, R.W., Jr., Moe, C.L., et al. (2015). Norovirus vaccine against experimental human GII.4 virus illness: a challenge study in healthy adults. J. Infect. Dis. *211*, 870–878.

Bok, K., Parra, G.I., Mitra, T., Abente, E., Shaver, C.K., Boon, D., Engle, R., Yu, C., Kapikian, A.Z., Sosnovtsev, S.V., et al. (2011). Chimpanzees as an animal model for human norovirus infection and vaccine development. Proc. Natl. Acad. Sci. USA *108*, 325–330.

Bootz, A., Karbach, A., Spindler, J., Kropff, B., Reuter, N., Sticht, H., Winkler, T.H., Britt, W.J., and Mach, M. (2017). Protective capacity of neutralizing and non-neutralizing antibodies against glycoprotein B of cytomegalovirus. PLoS Pathog. *13*, e1006601.

Bull, R.A., Tanaka, M.M., and White, P.A. (2007). Norovirus recombination. J. Gen. Virol. *88*, 3347–3359.

Burke, R.M., Shah, M.P., Wikswo, M.E., Barclay, L., Kambhampati, A., Marsh, Z., Cannon, J.L., Parashar, U.D., Vinjé, J., and Hall, A.J. (2019). The Norovirus Epidemiologic Triad: Predictors of Severe Outcomes in US Norovirus Outbreaks, 2009-2016. J. Infect. Dis. *219*, 1364–1372.

Cannon, J.L., Barclay, L., Collins, N.R., Wikswo, M.E., Castro, C.J., Magaña, L.C., Gregoricus, N., Marine, R.L., Chhabra, P., and Vinjé, J. (2017). Genetic and Epidemiologic Trends of Norovirus Outbreaks in the United States from 2013 to 2016 Demonstrated Emergence of Novel GII.4 Recombinant Viruses. J. Clin. Microbiol. *55*, 2208–2221.

Cardemil, C.V., Parashar, U.D., and Hall, A.J. (2017). Norovirus Infection in Older Adults: Epidemiology, Risk Factors, and Opportunities for Prevention and Control. Infect. Dis. Clin. North Am. *31*, 839–870.

Carmona-Vicente, N., Fernández-Jiménez, M., Ribes, J.M., Téllez-Castillo, C.J., Khodayar-Pardo, P., Rodríguez-Diaz, J., and Buesa, J. (2015). Norovirus infections and seroprevalence of genotype GII.4-specific antibodies in a Spanish population. J. Med. Virol. *87*, 675–682.

Chen, R., Neill, J.D., Noel, J.S., Hutson, A.M., Glass, R.I., Estes, M.K., and Prasad, B.V. (2004). Inter- and intragenus structural variations in caliciviruses and their functional implications. J. Virol. *78*, 6469–6479.

Chen, J., Zheng, Q., Hammers, C.M., Ellebrecht, C.T., Mukherjee, E.M., Tang, H.Y., Lin, C., Yuan, H., Pan, M., Langenhan, J., et al. (2017). Proteomic Analysis of Pemphigus Autoantibodies Indicates a Larger, More Diverse, and More Dynamic Repertoire than Determined by B Cell Genetics. Cell Rep. *18*, 237–247.

Collaborators, G.B.D.D.D.; GBD Diarrhoeal Diseases Collaborators (2017). Estimates of global, regional, and national morbidity, mortality, and aetiologies of diarrhoeal diseases: a systematic analysis for the Global Burden of Disease Study 2015. Lancet Infect. Dis. *17*, 909–948.

Costantini, V., Morantz, E.K., Browne, H., Ettayebi, K., Zeng, X.L., Atmar, R.L., Estes, M.K., and Vinjé, J. (2018). Human Norovirus Replication in Human Intestinal Enteroids as Model to Evaluate Virus Inactivation. Emerg. Infect. Dis. *24*, 1453–1464.

Debbink, K., Donaldson, E.F., Lindesmith, L.C., and Baric, R.S. (2012a). Genetic mapping of a highly variable norovirus GII.4 blockade epitope: potential role in escape from human herd immunity. J. Virol. *86*, 1214–1226.

Debbink, K., Lindesmith, L.C., Donaldson, E.F., and Baric, R.S. (2012b). Norovirus immunity and the great escape. PLoS Pathog. *8*, e1002921.

Debbink, K., Costantini, V., Swanstrom, J., Agnihothram, S., Vinje, J., Baric, R., and Lindesmith, L. (2013). Human norovirus detection and production, quantification, and storage of virus-like particles. Curr Protoc Microbiol *31*, 15K.1.1–15K.1.45.

Debbink, K., Lindesmith, L.C., and Baric, R.S. (2014a). The state of norovirus vaccines. Clin. Infect. Dis. *58*, 1746–1752.

Debbink, K., Lindesmith, L.C., Donaldson, E.F., Swanstrom, J., and Baric, R.S. (2014b). Chimeric GII.4 norovirus virus-like-particle-based vaccines induce broadly blocking immune responses. J. Virol. *88*, 7256–7266.

DeKosky, B.J., Kojima, T., Rodin, A., Charab, W., Ippolito, G.C., Ellington, A.D., and Georgiou, G. (2015). In-depth determination and analysis of the human paired heavy- and light-chain antibody repertoire. Nat. Med. *21*, 86–91.

DeKosky, B.J., Lungu, O.I., Park, D., Johnson, E.L., Charab, W., Chrysostomou, C., Kuroda, D., Ellington, A.D., Ippolito, G.C., Gray, J.J., and Georgiou, G. (2016). Large-scale sequence and structural comparisons of human naive and antigen-experienced antibody repertoires. Proc. Natl. Acad. Sci. USA *113*, E2636–E2645.

Diskin, R., Scheid, J.F., Marcovecchio, P.M., West, A.P., Jr., Klein, F., Gao, H., Gnanapragasam, P.N., Abadir, A., Seaman, M.S., Nussenzweig, M.C., and Bjorkman, P.J. (2011). Increasing the potency and breadth of an HIV antibody by using structure-based rational design. Science *334*, 1289–1293.

Emsley, P., and Cowtan, K. (2004). Coot: model-building tools for molecular graphics. Acta Crystallogr. D Biol. Crystallogr. *60*, 2126–2132.

Ettayebi, K., Crawford, S.E., Murakami, K., Broughman, J.R., Karandikar, U., Tenge, V.R., Neill, F.H., Blutt, S.E., Zeng, X.L., Qu, L., et al. (2016). Replication of human noroviruses in stem cell-derived human enteroids. Science *353*, 1387–1393.

Georgiou, G., Ippolito, G.C., Beausang, J., Busse, C.E., Wardemann, H., and Quake, S.R. (2014). The promise and challenge of high-throughput sequencing of the antibody repertoire. Nat. Biotechnol. *32*, 158–168.

Gostic, K.M., Ambrose, M., Worobey, M., and Lloyd-Smith, J.O. (2016). Potent protection against H5N1 and H7N9 influenza via childhood hemagglutinin imprinting. Science *354*, 722–726.

Green, K.Y., Lew, J.F., Jiang, X., Kapikian, A.Z., and Estes, M.K. (1993). Comparison of the reactivities of baculovirus-expressed recombinant Norwalk virus capsid antigen with those of the native Norwalk virus antigen in serologic assays and some epidemiologic observations. J. Clin. Microbiol. *31*, 2185–2191.

Hansman, G.S., Biertümpfel, C., Georgiev, I., McLellan, J.S., Chen, L., Zhou, T., Katayama, K., and Kwong, P.D. (2011). Crystal structures of GII.10 and GII.12 norovirus protruding domains in complex with histo-blood group antigens reveal details for a potential site of vulnerability. J. Virol. *85*, 6687–6701.

Hansman, G.S., Taylor, D.W., McLellan, J.S., Smith, T.J., Georgiev, I., Tame, J.R., Park, S.Y., Yamazaki, M., Gondaira, F., Miki, M., et al. (2012). Structural basis for broad detection of genogroup II noroviruses by a monoclonal antibody that binds to a site occluded in the viral particle. J. Virol. *86*, 3635–3646.

Harrington, P.R., Lindesmith, L., Yount, B., Moe, C.L., and Baric, R.S. (2002). Binding of Norwalk virus-like particles to ABH histo-blood group antigens is blocked by antisera from infected human volunteers or experimentally vaccinated mice. J. Virol. *76*, 12335–12343.

Havenar-Daughton, C., Sarkar, A., Kulp, D.W., Toy, L., Hu, X., Deresa, I., Kalyuzhniy, O., Kaushik, K., Upadhyay, A.A., Menis, S., et al. (2018). The human naive B cell repertoire contains distinct subclasses for a germline-targeting HIV-1 vaccine immunogen. Sci Transl Med *10*, eaat0381.

Henry Dunand, C.J., Leon, P.E., Huang, M., Choi, A., Chromikova, V., Ho, I.Y., Tan, G.S., Cruz, J., Hirsh, A., Zheng, N.Y., et al. (2016). Both Neutralizing and Non-Neutralizing Human H7N9 Influenza Vaccine-Induced Monoclonal Antibodies Confer Protection. Cell Host Microbe *19*, 800–813.

Ippolito, G.C., Hoi, K.H., Reddy, S.T., Carroll, S.M., Ge, X., Rogosch, T., Zemlin, M., Shultz, L.D., Ellington, A.D., Vandenberg, C.L., and Georgiou, G. (2012). Antibody Repertoires in Humanized NOD-scid-IL2Rgamma(null) Mice and Human B Cells Reveals Human-Like Diversification and Tolerance Checkpoints in the Mouse. PLoS One *7*, e35497.

Jiang, X., Matson, D.O., Ruiz-Palacios, G.M., Hu, J., Treanor, J., and Pickering, L.K. (1995). Expression, self-assembly, and antigenicity of a snow mountain agent-like calicivirus capsid protein. J. Clin. Microbiol. *33*, 1452–1455.

Kocher, J.F., Debbink, K., Lindesmith, L.C., Graham, R.L., Hugues, B., Goodwin, R.R., and Baric, R.S. (2018). Noroviurs Vaccines. In Plotkin's Vaccines, P.A.O, S.A. Plotkin, W.A. Orenstein, and K.M. Edwards, eds. (Philadelphia, PA: Elsevier), pp. 698–703.

Kolawole, A.O., Smith, H.Q., Svoboda, S.A., Lewis, M.S., Sherman, M.B., Lynch, G.C., Pettitt, B.M., Smith, T.J., and Wobus, C.E. (2017). Norovirus Escape from Broadly Neutralizing Antibodies Is Limited to Allostery-Like Mechanisms. MSphere *2*, e00334–e17.

Koromyslova, A.D., Morozov, V.A., Hefele, L., and Hansman, G.S. (2018). Human Norovirus Neutralized by a Monoclonal Antibody Targeting the Histo-Blood Group Antigen Pocket. J. Virol. *93*, e02174–e18.

Krissinel, E., and Henrick, K. (2007). Inference of macromolecular assemblies from crystalline state. J. Mol. Biol. *372*, 774–797.

Kwon, Y.D., Chuang, G.Y., Zhang, B., Bailer, R.T., Doria-Rose, N.A., Gindin, T.S., Lin, B., Louder, M.K., McKee, K., O'Dell, S., et al. (2018). Surface-Matrix Screening Identifies Semi-specific Interactions that Improve Potency of a Near Pan-reactive HIV-1-Neutralizing Antibody. Cell Rep. *22*, 1798–1809.

Lavinder, J.J., Wine, Y., Giesecke, C., Ippolito, G.C., Horton, A.P., Lungu, O.I., Hoi, K.H., DeKosky, B.J., Murrin, E.M., Wirth, M.M., et al. (2014). Identification and characterization of the constituent human serum antibodies elicited by vaccination. Proc. Natl. Acad. Sci. USA *111*, 2259–2264.

Lee, J., Boutz, D.R., Chromikova, V., Joyce, M.G., Vollmers, C., Leung, K., Horton, A.P., DeKosky, B.J., Lee, C.H., Lavinder, J.J., et al. (2016). Molecular-level analysis of the serum antibody repertoire in young adults before and after seasonal influenza vaccination. Nat. Med. *22*, 1456–1464.

Leroux-Roels, G., Cramer, J.P., Mendelman, P.M., Sherwood, J., Clemens, R., Aerssens, A., De Coster, I., Borkowski, A., Baehner, F., and Van Damme, P. (2018). Safety and Immunogenicity of Different Formulations of Norovirus Vaccine Candidate in Healthy Adults: A Randomized, Controlled, Double-Blind Clinical Trial. J. Infect. Dis. *217*, 597–607.

Lessler, J., Riley, S., Read, J.M., Wang, S., Zhu, H., Smith, G.J., Guan, Y., Jiang, C.Q., and Cummings, D.A. (2012). Evidence for antigenic seniority in influenza A (H3N2) antibody responses in southern China. PLoS Pathog. *8*, e1002802.

Lindesmith, L., Moe, C., Marionneau, S., Ruvoen, N., Jiang, X., Lindblad, L., Stewart, P., LePendu, J., and Baric, R. (2003). Human susceptibility and resistance to Norwalk virus infection. Nat. Med. *9*, 548–553.

Lindesmith, L.C., Donaldson, E.F., Lobue, A.D., Cannon, J.L., Zheng, D.P., Vinje, J., and Baric, R.S. (2008). Mechanisms of GII.4 norovirus persistence in human populations. PLoS Med. *5*, e31.

Lindesmith, L.C., Beltramello, M., Donaldson, E.F., Corti, D., Swanstrom, J., Debbink, K., Lanzavecchia, A., and Baric, R.S. (2012a). Immunogenetic mechanisms driving norovirus GII.4 antigenic variation. PLoS Pathog. *8*, e1002705.

Lindesmith, L.C., Debbink, K., Swanstrom, J., Vinjé, J., Costantini, V., Baric, R.S., and Donaldson, E.F. (2012b). Monoclonal antibody-based antigenic mapping of norovirus GII.4-2002. J. Virol. *86*, 873–883.

Lindesmith, L.C., Costantini, V., Swanstrom, J., Debbink, K., Donaldson, E.F., Vinjé, J., and Baric, R.S. (2013). Emergence of a norovirus GII.4 strain correlates with changes in evolving blockade epitopes. J. Virol. *87*, 2803–2813.

Lindesmith, L.C., Donaldson, E.F., Beltramello, M., Pintus, S., Corti, D., Swanstrom, J., Debbink, K., Jones, T.A., Lanzavecchia, A., and Baric, R.S. (2014). Particle conformation regulates antibody access to a conserved GII.4 norovirus blockade epitope. J. Virol. *88*, 8826–8842.

Lindesmith, L.C., Ferris, M.T., Mullan, C.W., Ferreira, J., Debbink, K., Swanstrom, J., Richardson, C., Goodwin, R.R., Baehner, F., Mendelman, P.M., et al. (2015). Broad blockade antibody responses in human volunteers after immunization with a multivalent norovirus VLP candidate vaccine: immunological analyses from a phase I clinical trial. PLoS Med. *12*, e1001807.

Lindesmith, L.C., Mallory, M.L., Jones, T.A., Richardson, C., Goodwin, R.R., Baehner, F., Mendelman, P.M., Bargatze, R.F., and Baric, R.S. (2017). Impact of Pre-exposure History and Host Genetics on Antibody Avidity Following Norovirus Vaccination. J. Infect. Dis. *215*, 984–991.

Lindesmith, L.C., Brewer-Jensen, P.D., Mallory, M.L., Debbink, K., Swann, E.W., Vinjé, J., and Baric, R.S. (2018a). Antigenic Characterization of a Novel Recombinant GII.P16-GII.4 Sydney Norovirus Strain With Minor Sequence Variation Leading to Antibody Escape. J. Infect. Dis. *217*, 1145–1152.

Lindesmith, L.C., Mallory, M.L., Debbink, K., Donaldson, E.F., Brewer-Jensen, P.D., Swann, E.W., Sheahan, T.P., Graham, R.L., Beltramello, M., Corti, D., et al. (2018b). Conformational Occlusion of Blockade Antibody Epitopes, a Novel Mechanism of GII.4 Human Norovirus Immune Evasion. MSphere *3*, e00518–e17.

LoBue, A.D., Lindesmith, L., Yount, B., Harrington, P.R., Thompson, J.M., Johnston, R.E., Moe, C.L., and Baric, R.S. (2006). Multivalent norovirus vaccines induce strong mucosal and systemic blocking antibodies against multiple strains. Vaccine *24*, 5220–5234.

Mallory, M.L., Lindesmith, L.C., Graham, R.L., and Baric, R.S. (2019). GII.4 Human Norovirus: Surveying the Antigenic Landscape. Viruses *11*, 177.

Malm, M., Uusi-Kerttula, H., Vesikari, T., and Blazevic, V. (2014). High serum levels of norovirus genotype-specific blocking antibodies correlate with protection from infection in children. J. Infect. Dis. *210*, 1755–1762.

McCoy, A.J., Grosse-Kunstleve, R.W., Adams, P.D., Winn, M.D., Storoni, L.C., and Read, R.J. (2007). Phaser crystallographic software. J. Appl. Cryst. *40*, 658–674.

McDaniel, J.R., DeKosky, B.J., Tanno, H., Ellington, A.D., and Georgiou, G. (2016). Ultra-high-throughput sequencing of the immune receptor repertoire from millions of lymphocytes. Nat. Protoc. *11*, 429–442.

McLellan, J.S., Pancera, M., Carrico, C., Gorman, J., Julien, J.P., Khayat, R., Louder, R., Pejchal, R., Sastry, M., Dai, K., et al. (2011). Structure of HIV-1 gp120 V1/V2 domain with broadly neutralizing antibody PG9. Nature *480*, 336–343.

Mroczek, E.S., Ippolito, G.C., Rogosch, T., Hoi, K.H., Hwangpo, T.A., Brand, M.G., Zhuang, Y., Liu, C.R., Schneider, D.A., Zemlin, M., et al. (2014). Differences in the composition of the human antibody repertoire by B cell subsets in the blood. Front. Immunol. *5*, 96.

Nurminen, K., Blazevic, V., Huhti, L., Räsänen, S., Koho, T., Hytönen, V.P., and Vesikari, T. (2011). Prevalence of norovirus GII-4 antibodies in Finnish children. J. Med. Virol. *83*, 525–531.

Otwinowski, Z., and Minor, W. (1997). Processing of X-ray diffraction data collected in oscillation mode. Methods Enzymol. *276*, 307–326.

Park, J.S., Yeom, J.S., Seo, J.H., Lim, J.Y., Park, C.H., Woo, H.O., Youn, H.S., Jun, J.S., Park, J.H., Ko, G.H., et al. (2015). Immunohistochemical Expressions of MUC2, MUC5AC, and MUC6 in Normal, Helicobacter pylori Infected and Metaplastic Gastric Mucosa of Children and Adolescents. Helicobacter *20*, 260–268.

Parra, G.I., Bok, K., Taylor, R., Haynes, J.R., Sosnovtsev, S.V., Richardson, C., and Green, K.Y. (2012). Immunogenicity and specificity of norovirus Consensus GII.4 virus-like particles in monovalent and bivalent vaccine formulations. Vaccine *30*, 3580–3586.

Patel, M.M., Widdowson, M.A., Glass, R.I., Akazawa, K., Vinjé, J., and Parashar, U.D. (2008). Systematic literature review of role of noroviruses in sporadic gastroenteritis. Emerg. Infect. Dis. *14*, 1224–1231.

Pei, J., and Grishin, N.V. (2001). AL2CO: calculation of positional conservation in a protein sequence alignment. Bioinformatics *17*, 700–712.

Petrignani, M., Verhoef, L., de Graaf, M., Richardus, J.H., and Koopmans, M. (2018). Chronic sequelae and severe complications of norovirus infection: A systematic review of literature. J. Clin. Virol. *105*, 1–10.

Pettersen, E.F., Goddard, T.D., Huang, C.C., Couch, G.S., Greenblatt, D.M., Meng, E.C., and Ferrin, T.E. (2004). UCSF Chimera–a visualization system for exploratory research and analysis. J. Comput. Chem. *25*, 1605–1612.

Pogan, R., Dülfer, J., and Uetrecht, C. (2018). Norovirus assembly and stability. Curr. Opin. Virol. *31*, 59–65.

Prasad, B.V., Hardy, M.E., Dokland, T., Bella, J., Rossmann, M.G., and Estes, M.K. (1999). X-ray crystallographic structure of the Norwalk virus capsid. Science *286*, 287–290.

Reeck, A., Kavanagh, O., Estes, M.K., Opekun, A.R., Gilger, M.A., Graham, D.Y., and Atmar, R.L. (2010). Serological correlate of protection against norovirus-induced gastroenteritis. J. Infect. Dis. *202*, 1212–1218.

Shanker, S., Choi, J.M., Sankaran, B., Atmar, R.L., Estes, M.K., and Prasad, B.V. (2011). Structural analysis of histo-blood group antigen binding specificity in a norovirus GII.4 epidemic variant: implications for epochal evolution. J. Virol. *85*, 8635–8645.

Shanker, S., Czakó, R., Sapparapu, G., Alvarado, G., Viskovska, M., Sankaran, B., Atmar, R.L., Crowe, J.E., Jr., Estes, M.K., and Prasad, B.V. (2016). Structural basis for norovirus neutralization by an HBGA blocking human IgA antibody. Proc. Natl. Acad. Sci. USA *113*, E5830–E5837.

Siebenga, J.J., Vennema, H., Renckens, B., de Bruin, E., van der Veer, B., Siezen, R.J., and Koopmans, M. (2007). Epochal evolution of GGII.4 norovirus capsid proteins from 1995 to 2006. J. Virol. *81*, 9932–9941.

Singh, B.K., Leuthold, M.M., and Hansman, G.S. (2015). Human noroviruses' fondness for histo-blood group antigens. J. Virol. *89*, 2024–2040.

Snijder, J., Ortego, M.S., Weidle, C., Stuart, A.B., Gray, M.D., McElrath, M.J., Pancera, M., Veesler, D., and McGuire, A.T. (2018). An Antibody Targeting the Fusion Machinery Neutralizes Dual-Tropic Infection and Defines a Site of Vulnerability on Epstein-Barr Virus. Immunity *48*, 799–811.e9.

Tan, M., and Jiang, X. (2011). Norovirus-host interaction: multi-selections by human histo-blood group antigens. Trends Microbiol. *19*, 382–388.

Treanor, J.J., Atmar, R.L., Frey, S.E., Gormley, R., Chen, W.H., Ferreira, J., Goodwin, R., Borkowski, A., Clemens, R., and Mendelman, P.M. (2014). A novel intramuscular bivalent norovirus virus-like particle vaccine candidate–reactogenicity, safety, and immunogenicity in a phase 1 trial in healthy adults. J. Infect. Dis. *210*, 1763–1771.

Voss, N.R., and Gerstein, M. (2010). 3V: cavity, channel and cleft volume calculator and extractor. Nucleic Acids Res. *38*, W555–W562.

Williams, L.D., Ofek, G., Schatzle, S., McDaniel, J.R., Lu, X., Nicely, N.I., Wu, L., Lougheed, C.S., Bradley, T., Louder, M.K., et al. (2017). Potent and broad HIV-neutralizing antibodies in memory B cells and plasma. Sci Immunol *2*, eaal2200.

Word, J.M., Lovell, S.C., LaBean, T.H., Taylor, H.C., Zalis, M.E., Presley, B.K., Richardson, J.S., and Richardson, D.C. (1999). Visualizing and quantifying molecular goodness-of-fit: small-probe contact dots with explicit hydrogen atoms. J. Mol. Biol. *285*, 1711–1733.

Zheng, D.P., Ando, T., Fankhauser, R.L., Beard, R.S., Glass, R.I., and Monroe, S.S. (2006). Norovirus classification and proposed strain nomenclature. Virology *346*, 312–323.

CellPress

STAR★METHODS

KEY RESOURCES TABLE

REAGENT or RESOURCE	SOURCE	IDENTIFIER
Antibodies		
Goat anti-human IgG Fc−peroxidase antibody	Invitrogen	Cat# A18817
Donkey anti-rabbit IgG-HRP	GE Healthcare	Cat# NA934; RRID: AB_772206
Bacterial and Virus Strains		
E.coli One Shot TOP10	Invitrogen	Cat# C404010
E.coli BL21 (DE3)	Agilent Technologies	Cat# 200131
Biological Samples		
Rabbit anti-norovirus capsid protein polyclonal serum	CoCalico Biologicals Custom Synthesis	N/A
GII.P16-GII.4 Sydney	Positive stool sample	N/A
Chemicals, Peptides, and Recombinant Proteins		
GI.1 Virus like particles	R. Baric, UNC-CH	GenBank: AFJ38516.1
GI.3 Virus like particles	R. Baric, UNC-CH	GenBank: AFK75851.1
GI.4 Virus like particles	R. Baric, UNC-CH	GenBank: AFK75852.1
GII.4 1987 Virus like particles	R. Baric, UNC-CH	GenBank: AAK50355.1
GII.4 1997 Virus like particles	R. Baric, UNC-CH	GenBank: AFJ04707.1
GII.4 2002 Virus like particles	R. Baric, UNC-CH	GenBank: AFJ04708.1
GII.4 2006b Virus like particles	R. Baric, UNC-CH	GenBank: AFJ04709.1
GII.4 2009 Virus like particles	R. Baric, UNC-CH	GenBank: ADD10375.1
GII.4 2012 Virus like particles	R. Baric, UNC-CH	GenBank: AFV08795.1
GII.4 2015 Virus like particles	R. Baric, UNC-CH	GenBank: APA31970.1
GII.1 Virus like particles	R. Baric, UNC-CH	GenBank: AFS33555.1
GII.2 Virus like particles	R. Baric, UNC-CH	GenBank: AAN08112.1
GII.3 Virus like particles	R. Baric, UNC-CH	GenBank: AFK75854.1
GII.14 Virus like particles	R. Baric, UNC-CH	GenBank: AAN05735.1
GII.17 Virus like particles	R. Baric, UNC-CH	GenBank: AKB94547.1
GII.4c Virus like particles	This study	GenBank: MK614455
GII.4 2006a.581 Virus like particles	R. Baric, UNC-CH	GenBank: MK614084
GII.4 2006a.581.A4A Virus like particles	This study	GenBank: MK614085
GII.4 2006a.581.A4B Virus like particles	This study	GenBank: MK614086
GII.4 1987.2012A Virus like particles	This study	GenBank: MK614081
GII.4 1987.2012D Virus like particles	This study	GenBank: MK614082
GII.4 1987.2012E Virus like particles	This study	GenBank: MK614083
Pig gastric mucin	Sigma-Aldrich	Cat# M1778
Matrigel	Corning	Cat# 356231
IntestiCult Organoid Growth Medium (Human)	STEMCELL Technologies	Cat# 06010
Y-27632	Sigma-Aldrich	Cat# Y0503-1MG
Advanced Dulbecco Modified Eagle Medium (DMEM)/F12	Invitrogen	Cat# 12634-028
GlutaMAX	Invitrogen	Cat# 35050-061
Penicillin/Streptomycin	Invitrogen	Cat# 15140-122
1M HEPES	Invitrogen	Cat# 15630-080
B27 supplement	Invitrogen	Cat# 17504-044
N2 supplement	Invitrogen	Cat# 17502-048
mouse epidermal growth factor	Invitrogen	Cat# PMG8043

(*Continued on next page*)

Continued

REAGENT or RESOURCE	SOURCE	IDENTIFIER
n-Acetyl-L-cysteine	Sigma-Aldrich	Cat# A9165-5G
[Leu15]-Gastrin	Sigma-Aldrich	Cat# G9145
Noggin media	Ettayebi et al 2016	N/A
A83-01	Tocris	Cat# 2939
DPBS, no calcium, no magnesium	Invitrogen	Cat# 14190-250
Sodium glycochenodeoxycholate	Sigma-Aldrich	Cat# G0759-100MG
Collagen from human placenta (collagen type IV)	Sigma-Aldrich	Cat# C5533-5MG
C2 Ceramide	Santa Cruz Biotechnology	Cat# sc-201375A
5 M Ethylenediaminetetraacetic (EDTA)	Invitrogen	Cat# 15575-020
0.05% Trypsin/0.5mM EDTA	Gibco	Cat# 25300054
Fetal bovine serum certified	Life Technologies	Cat# 16000-044
cOmplete, EDTA-free Protease Inhibitor Cocktail	Sigma-Aldrich	Cat# 5056489001
Lysozyme from chicken egg white	Sigma-Aldrich	Cat# L6876
cOmplete His-Tag Purification Resin	Sigma-Aldrich	Cat# 5893801001
HRV-3C Protease	Sigma-Aldrich	Cat# SAE0045
Turbo293 Transfection Reagent	Speed Biosystems	Cat# PXX1002
AbBooster, Antibody Expression Enhancer	Abi Scientific	Cat# PB2668
Expi293 Expression Medium	Thermo Fisher Scientific	Cat# A1435101
GE MabSelect Protein A Resin	Sigma-Aldrich	Cat# GE17-5199-01
Pierce IgG Elution Buffer	Thermo Fisher Scientific	Cat# 21004
Polyethylene Glycol 4000	Rigaku	Cat# 1008059
Tris-HCl buffer	Rigaku	Cat# HR2-237
0.1 M L-Proline	Hampton Research	Cat# HR2-428-26
Polyethylene Glycol 8000	Rigaku	Cat# 1008062
CHES/Sodium Hydroxide	Rigaku	Cat#1008158
Critical Commercial Assays		
mMESSAGE MACHINE T7 Transcription kit	Invitrogen	Cat# AM1344
Deoxyribonuclease I	Sigma Aldrich	Cat# D4513-1VL
TRIzol	Thermo Fisher Scientific	Cat# 15596-026
Rneasy Kit	Qiagen	Cat# 74004
SuperScript II	Invitrogen	Cat# 11904-018
Human Memory B Cell Isolation Kit	Miltenyi	Cat# 130-094-350
Oligo d(T)25 magnetic beads	NEB	Cat# S1419S
One step fast qRT-PCR solution	VWR	Cat# 95039-940
N-hydroxysuccinimide (NHS)-agarose beads	Thermo Fisher Scientific	Cat# 26197
Melon gel purification	Thermo Fisher Scientific	Cat# 45212
Tris(2-carboxyethyl)phosphine	Thermo Fisher Scientific	Cat# 77720
Iodoacetamide	Sigma Aldrich	Cat# I1149-5G
Trifluoroethanol	Thermo Fisher Scientific	Cat# AC13975-1000
Trypsin Gold	Thermo Fisher Scientific	Cat# PR-V5280
5x MagMAX - 96 Viral RNA Isolation Kit	Applied Biosystems	Cat# AMB1836-5
AgPath-ID One-Step RT-PCR	Applied Biosystems	Cat# 4387391
Deposited Data		
Crystal structure of GII.4.2002 P domain in complex with A1227 Fab	This paper	PDB: 6N81
Crystal structure of GII.4.2002 P domain in complex with A1431 Fab	This paper	PDB: 6N8D
GII.4c Virus like particles	This study	GenBank: MK614455

(*Continued on next page*)

Continued

REAGENT or RESOURCE	SOURCE	IDENTIFIER
GII.4 2006a.581 Virus like particles	R. Baric, UNC-CH	GenBank: MK614084
GII.4 2006a.581.A4A Virus like particles	This study	GenBank: MK614085
GII.4 2006a.581.A4B Virus like particles	This study	GenBank: MK614086
GII.4 1987. 2012A Virus like particles	This study	GenBank: MK614081
GII.4 1987. 2012D Virus like particles	This study	GenBank: MK614082
GII.4 1987. 2012E Virus like particles	This study	GenBank: MK614083
Experimental Models: Cell Lines		
BHK cell line	ATCC	Cat# CCL-10
Human intestinal enteroids (J3 cell line)	Ettayebi et al., 2016	N/A
Human: Expi293F cells	Thermo Fisher Scientific	Cat# A14527
Oligonucleotides		
Cog 1F (5′-CGYTGGATGCGITTYCATGA-3′)	Cannon et al., 2017	N/A
Cog 1R (5′-CTTAGACGCCATCATCATTYAC-3′)	Cannon et al., 2017	N/A
Probe Ring 1E (5′-FAM-TGGACAGGRGAYCGC-MGBNFQ-3′)	Cannon et al., 2017	N/A
MS2.F (5′-TGGCACTACCCCTCTCCGTATTCACG-3′)	Cannon et al., 2017	N/A
MS2.R (5′-GTACGGGCGACCCCACGATGAC-3′)	Cannon et al., 2017	N/A
Probe MS2.P (5′-HEX-CACATCGATAGATCAAGG TGCCTACAAGC-BHQ1-3′)	Cannon et al., 2017	N/A
Cog 2F (5′-CARGARBCNATGTTYAGRTGGATGAG-3′)	Cannon et al., 2017	N/A
Cog 2R (5′-TCGACGCCATCTTCATTCACA-3′)	Cannon et al., 2017	N/A
Probe Ring 2 (5′-Cy5-TGGGAGGGCGATCGCAATCT-BHQ2-3′)	Cannon et al., 2017	N/A
Recombinant DNA		
pVR21	R. Baric, UNC	N/A
pUC57 with norovirus capsid inserted	BioBasic Custom Synthesis	N/A
pVRC8400 Vector	NIH/VRC	N/A
pMal-C5X plasmid	New England Biolabs	Cat# N8108S
Software and Algorithms		
Prism	https://www.graphpad.com/scientific-software/prism/	RRID: SCR_002798
Sequest, Percolator on Proteome Discoverer 1.4	Thermo Fisher Scientific	https://www.thermofisher.com/order/catalog/product/OPTON-30795
HKL2000	HKL Research	http://www.hkl-xray.com/
Phenix	Adams et al., 2004	https://sbgrid.org/software/
Coot	Emsley and Cowtan, 2004	https://sbgrid.org/software/
Pymol	Schrödinger	https://pymol.org/2
Chimera	Pettersen et al., 2004	https://www.cgl.ucsf.edu/chimera/
AL2CO	Pei and Grishin, 2001	http://prodata.swmed.edu/al2co/al2co.php
PDBePISA	Krissinel and Henrick, 2007	http://www.ebi.ac.uk/pdbe/pisa/
Other		
KingFisher Flex Purification System	ThermoFisher Scientific	Cat# 5400610
GII.4 Sydney RNA transcripts	CDC	N/A
7500 Real-Time PCR System	Applied Biosystems	Cat# 4351104

CONTACT FOR REAGENT AND RESOURCE SHARING

Further information and requests for resources and reagents should be directed to and will be fulfilled by the Lead Contact, Ralph Baric (rbaric@email.unc.edu). All human samples remain property of Takeda Vaccines.

EXPERIMENTAL MODEL AND SUBJECT DETAILS

Humans Subjects

Serum and PBMC samples

This study used de-identified human samples under University of North Carolina institutional review board exemption approval #11-0883. Serum and PBMC samples were collected from two female ages 25 and 46 and one male age 30 participants in a study conducted by Takeda Vaccines Inc. (Deerfield, IL) (Treanor et al., 2014). Participants in Group A1 received an intramuscular injection of 5 μg of both GI.1 and GII.4c VLP adjuvanted with 3-O-deacylated monophosphoryl lipid A (MPL, GlaxoSmithKline) and alhydrogel on day 0 (dose 1) followed by an identical booster vaccination on day 28 (dose 2). This study was done under IRB approval and subjects provided informed consent. The trial was registered at ClinicalTrials.gov (NCT01168401).

Cell lines

BHK -21 cells (ATCC CCL-10, derived from unsexed hamsters) were grown at 37°C in 5% CO_2 in Minimum Essential Medium with Earles salts and L-glutamine (Gibco) supplemented with 7% fetal Clone II (Hyclone), non-essential amino acids (Gibco), sodium pyruvate (Gibco) and Antibiotic-Antimycotic (Gibco).

Microbes

E coli Top 10 with pVR21 plasmid containing norovirus capsid genes were propagated at 30°C in the presence of 0.1mg/ml carbenicillin in Luria-Bertani broth. For full IgG expression and characterization, VH and VL plasmids were transfected into 30 mL cultures of Expi293F cells (Invitrogen) at a 1:2 ratio and incubated at 37°C and 8% CO_2 for 7 days.

For protein crystallization, P-domains were transfected into *E.coli* BL21 (DE3) (Agilent Technologies) and plated onto Luria-Bertani plates with 50μg/mL Ampicillin (LB-Amp). VH and VL chains were cotransfected into Expi293F cells (ThermoFisher) using a 3:1 ratio of Turbo293 transfection reagent (Speed Biosystem) to plasmid. Cells were incubated at 37°C, 5% CO_2, 130 rpm shaking, 70% humidity for 18 h and subsequently boosted with 80mL of AbBooster (Abi Scientific).

METHOD DETAILS

Ten percent stool filtrate

This investigation was determined by CDC to be public health non-research, and therefore was not subject to institutional review board review. Ten percent stool suspension was prepared by adding 0.5 g of GII.P16-GII.4 Sydney positive whole stool to 4.5 mL of PBS. The stool suspension was vortexed for 30 seconds, kept at room temperature for 5 min and vortexed again. Sample was sonicated and solids were removed by centrifugation for 10 min at 10,000 x g. The supernatant was serially filtered through 5 μm, 1 μm, 0.45 μm, and 0.22 μm filters. The resulting 10% stool filtrate was aliquoted and stored at −70°C.

Human intestinal enteroid culture (HIE)

HIE cells were kindly provided by Dr. Mary K. Estes, Baylor College of Medicine, Houston, Texas. Adult (female) secretor positive jejunal HIE cultures were grown as undifferentiated 3D cultures as described previously with minor modifications (Costantini et al., 2018; Ettayebi et al., 2016). Briefly, HIEs were recovered from liquid nitrogen (LN_2), suspended in 20 μl of Matrigel (Corning), plated in a single well of a 24 well plate, and grown as 3D cultures in 500 μL IntestiCult Organoid Growth Medium (Human) (STEMCELL Technologies) supplemented with 10 μM Y-27632 (Sigma-Aldrich). Media was refreshed every other day. Highly dense 3D cultures were passaged once per week. To passage, HIE were taken up in ice-cold complete medium without growth factors (CMGF-), broken up by repeating pipetting cycles, suspended in Matrigel (Corning), and plated in 24 well plates. CMGF- compromises Advanced Dulbecco Modified Eagle Medium (DMEM)/F12 supplemented with 1% GlutaMAX, 1% Penicillin/Streptomycin, 1% 1M HEPES. If single cell suspension was required, HIE were taken up in ice-cold 0.5mM EDTA and broken up by repeating pipetting cycles. After centrifugation (200 x g for 5 min at 4°C), single cell suspension was obtained upon treatment of the HIE with 0.05% Trypsin/0.5mM EDTA (Invitrogen) for 3–4 min at 37°C. Trypsin was then inactivated by adding CMGF- /10% FBS to the cell suspension. Cell suspensions were prepared by pipetting with a P1000 pipet and passing the cells through a 40 μm cell strainer. The cells were pelleted for 5 min at 400 x *g*, suspended in 100 μl of in IntestiCult Organoid Growth Medium (Human) (STEMCELL Technologies) supplemented with 10 μM Y-27632 (Sigma-Aldrich) and plated as undifferentiated monolayers in collagen IV (Sigma-Aldrich) pre-coated 96 well plates. After 24 h, culture medium was replaced with differentiation medium to induce cell differentiation. Differentiation media compromises CMGF- ,1:100 B27 supplement, 1:50 N2 supplement, 1:1000 mouse epidermal growth factor (all Invitrogen), 1 mM n-Acetyl-L-cysteine, 10 nM [Leu15]-Gastrin, 10 μM Y-27632 (all Sigma-Aldrich), Noggin 20%, (conditioned home-made medium), 500 nM A83-01 (Tocris). Cells were differentiated for 4 days. Media was refreshed every other day.

High-throughput sequencing of BCR transcripts

Frozen PBMCs were thawed at 37°C, resuspended in RPMI-1640 (Fisher) supplemented with 10% Fetal Bovine Serum (FBS) and 20 U/mL deoxyribonuclease I (Roche), concentrated by centrifugation (300 x g for 15 min at 20°C), and allowed to recover in 4 mL media at 37°C for 30 min. The cells were then split: half were dedicated to BCR heavy chain transcript sequencing, and half were used to identify the native VH/VL repertoire.

VH Sequencing

Cells were diluted with 10 mL of cold buffer (phosphate buffered saline supplemented with 0.5% BSA and 2 mM EDTA) and pelleted via centrifugation (300 x g for 15 min at 4°C). The supernatant was decanted, and the cells were resuspended in 1mL of TRIzol reagent (Thermo Fisher). RNA was extracted using RNeasy (Qiagen), and first strand cDNA was synthesized from 500 ng total RNA using SuperScript II (Invitrogen). IgM and IgG heavy chain repertoires were amplified using a multiplex primer set (Table S3) under the following conditions: 2 min at 94°C; 4 cycles of 94°C for 30 s, 50°C for 30 s, 72°C for 30 s; 4 cycles of 94°C for 30 s, 55°C for 30 s, 72°C for 30 s; 22 cycles of 94°C for 30 s, 63°C for 30 s, 72°C for 30 s; 72°C for 7 min; hold at 4°C (Ippolito et al., 2012) and sequenced by 2x300 paired-end Illumina MiSeq.

VH/VL Sequencing

Total B cells were isolated from PBMCs using the Human Memory B Cell Isolation Kit (Miltenyi Biotec) with an LD column according to the manufacturer's instructions. B cells were then coemulsified with oligo d(*T*)25 magnetic beads (New England Biolabs) and lysis buffer (100 mM Tris pH 7.5, 500 mM LiCl, 10 mM EDTA, 1% lithium dodecyl sulfate, and 5 mM dithiothreitol) using a custom-designed flow focusing device as described (McDaniel et al., 2016). The magnetic beads were washed, resuspended in One step fast qRT-PCR solution (VWR) supplemented with an overlap extension VH and VL primer set as described (McDaniel et al., 2016), emulsified, and subjected to overlap-extension RT-PCR under the following conditions: 30 min at 55 °C followed by 2 min at 94 °C; 4 cycles of 94 °C for 30 s, 50 °C for 30 s, 72 °C for 2 min; 4 cycles of 94 °C for 30 s, 55 °C for 30 s, 72 °C for 2 min; 32 cycles of 94 °C for 30 s, 60 °C for 30 s, 72 °C for 2 min; 72 °C for 7 min; hold at 4 °C. VH/VL amplicons were then extracted from the emulsions, amplified using nested PCR, and sequenced by 2x300 Illumina MiSeq.

Isolation of HuNoV specific antibodies

HuNoV VLP (GII.4c, 1 mg) was immobilized on 250 mg N-hydroxysuccinimide (NHS)-agarose beads (Thermo Fisher) by overnight rotation at 4°C. The conjugated resin was washed with PBS, and unreacted NHS groups were blocked with 1M ethanolamine, pH 8.3, for 20 min at RT. The beads were washed with PBS, packed into a 5 mL column, washed with 10 column volumes (cv) of 4M urea in PBS, and finally washed with 20 cv PBS. Total IgG was isolated from each serum sample using melon gel purification (Thermo Fisher) according to the manufacturer's instructions. IgG was then passed over the affinity column in gravity flow mode to capture GII.4c VLP-specific antibodies. Flow through was collected and reapplied to the column five times. The column was washed with PBS with 2 cv followed by 1 cv of water before elution in 12-1 mL fractions of 5% formic acid. 10 μL of each elution fraction was neutralized with 100 μl of 25x PBS in 2% milk and analyzed via ELISA against GII.4c VLP (antibodies bound to immobilized GII.4c were detected using goat anti-human IgG Fc−peroxidase antibody (Invitrogen)). Fractions containing antigen-specific antibodies were pooled, concentrated under vacuum, and resuspended into 50 μL of 100 mM Tris-pH 8.

Sample preparation and LC-MS/MS analysis

For each sample, the flow through and antigen enriched elution were denatured in 1:1 (v/v) trifluoroethanol, reduced with 5 μL tris(2-carboxyethyl)phosphine (TCEP, 110 mM) in the dark at 55°C for 45 min, and alkylated with 32 mM iodoacetamide by incubating in the dark for 30 min. Samples were diluted 10x into 50 mM Tris (pH 8.0) and digested with trypsin (1:10 trypsin/protein) for 5 h at 37°C. Formic acid (0.1%) was used to quench the reaction. The solution was then concentrated, desalted using a C18 Hypersep SpinTip (Thermo) according to the manufacturer's instructions, and submitted for LC-MS/MS.

Peptides were separated by reversed-phase chromatography (Dionex UltiMate 3000 RSLCnano System with Dionex Acclaim PepMap RSLC C18 column, Thermo Scientific) and analyzed online by nano–electrospray ionization–MS/MS on Orbitrap Velos Pro (Thermo Scientific). MS1 scans were collected in the orbitrap at a resolution of 60,000 Å, and the ions with >+1 charge were fragmented by collision-induced dissociation with up to 20 MS2 spectra collected per MS1.

SEQUEST (Proteome Discoverer 1.4, Thermo Scientific) with previously described settings (Williams et al., 2017) was used to search the spectra against a patient-specific protein database constructed from the full-length VH and VL sequences, Ensembl human protein-coding sequences, and common contaminants (maxquant.org). PSMs were filtered with Percolator (Proteome Discoverer 1.4) at a false discovery rate of <1% and filtered for average mass deviations <1.5 parts per million. Peptides mapping to the CDR-H3 region of unique antibody lineages were grouped, and the relative abundances of the corresponding peptide matches were determined by the sum of the extracted ion chromatograms (XIC) of the respective precursor ions. Relative abundances were multiplied by the patient specific anti-GII.4c titer to calculate the amount of GII.4c specific clonotypes in serum.

Antibody expression and purification

Selected antibody sequences were purchased as gBlocks gene fragments (Integrated DNA Technologies) and cloned into a customized pcDNA3.4 vector (Invitrogen) containing human IgG1 Fc regions. VH and VL plasmids were transfected into 30 mL cultures of Expi293F cells (Invitrogen) at a 1:2 ratio and incubated at 37°C and 8% CO_2 for 7 days. The supernatant containing secreted antibodies was collected following centrifugation (1000 g for 10 min at 4°C), neutralized, and filtered. Antibodies were isolated using Protein G Plus agarose (Pierce Thermo Fisher Scientific) affinity chromatography, washed with 20 column volumes of PBS, eluted with 100 mM glycine-HCl pH 2.7, and neutralized with 1 M Tris-HCl pH 8.0. The antibodies were then concentrated and buffer exchanged into PBS using 10,000 MWCO Vivaspin centrifugal spin columns (Sartorius).

Production of Virus Like Particles

For production of the VLP for this study, HuNoV ORF2 genes were synthesized by Bio Basic Inc (Markham, ON) with ApaI and AcsI restriction sites for direct insertion into the VEE replicon vector pVR21. The sequence-verified pDNA was propagated in Ecoli Top 10 (Invitrogen). pDNA was purified with QIAprep Miniprep kit (Qiagen). Purified pDNA was linearized with NotI, agarose gel purified with QIAquick Gel Extraction kit (Qiagen) and mRNA generated using mMESSAGE mMachine T7 transcription kit (Invtirogen). Message RNA was electroplated into BHK cells (ATCC CCL-10) for production of VLP (Agnihothram et al., 2018; Debbink et al., 2013). After 27 h, cells were washed and lysed in 1% Triton X-100 in PBS plus cOmplete EDTA-free protease inhibitor cocktail (Roche) and cell lysates layered above a cushion of 40% sucrose in PBS. VLP were purified into the cushion by ultracentrifugation at 120,000 x g for 75 min and stored at -80°C in 40% sucrose/PBS. Protein concentration was determined by BCA assay (Pierce). VLPs were verified for particle integrity by ligand and antibody binding and visualization by electron microscopy of ~40nm particles.

Antibody functional assays

EIA and blockade antibody assays were performed at described (Lindesmith et al., 2018a) at 0.25 μg/ml VLP. EIA plates were coated with VLP in PBS for 4 h and blocked over night at 4°C in 5% dry milk in PBS-0.05% Tween-20 before the addition of decreasing two-fold serial dilutions of mAb at 37°C. Bound mAb was detected by anti-human IgG-HRP (GE Healthcare) and color developed with 1-Step Ultra TMB ELISA HRP substrate solution (Thermo-Fisher). Similarly, VLPs were pretreated with decreasing concentrations of mAb for 1 h and added to pig gastric mucin type III (Sigma Aldrich, St. Louis, MO) coated plates for 1 h. Bound VLP was detected by anti-VLP rabbit hyperimmune sera followed by anti-rabbit IgG-HRP (GE Healthcare) and color developed as above. Percent control binding is defined as the binding in the presence of antibody pretreatment compared to the binding in the absence multiplied by 100.

P domain expression and purification

The gene for the P-domain of HuNoV GII.4.2002 (accession number: AFJ04708) corresponding to amino acids 225-530 was synthesized by GenScript and codon optimized for *E. coli* expression. The P-domain gene was cloned in pMal-C5X plasmid (NEB) with a HRV-3C cleavage site between the MBP-6xHis and the P-domain (Nucleotide sequence: CATCACCACCACCAT CACGGTTCTGGTCTGGAAGTTC-TGTTCCAGGGGCCCTCC). The plasmid was transformed into *E.coli* BL21 (DE3) (Agilent Technologies) and plated onto Luria-Bertani plates with 50μg/mL Ampicillin (LB-Amp). A single colony was used to inoculate 100mL of LB-Amp. After incubation for 18 h at 37°C and 210 rpm shaking, 10 mL of culture was used to inoculate 1L of LB-Amp at 37°C and 210 rpm shaking. When OD_{600} reached 0.7, flasks were cooled for 30 min in ice/water bath. Flasks were subsequently incubated at 18°C and 210 rpm shaking for 30 min before induction with 0.5mM IPTG (Isopropyl β-D-1-thiogalactopyranoside). After 18 h, cells were harvested by centrifugation at 10,000 x g for 20 min at 20°C. Pellets from 1L cell culture were resuspended in 40mL of PBS with addition of 150mM of NaCl and flash frozen in liquid nitrogen. Cell pellets were stored at -80°C until further use.

Pellets from 1L of cell culture were thawed on ice and supplemented with one tablet of protease inhibitors (Roche cOmplete EDTA-free cocktail) and 1mg/mL of Lysozyme (Sigma-Aldrich). Cell slurry was kept on ice and sonicated with a Branson Sonifier SFX150 equipped with micro-tip (5 seconds ON, 30 seconds OFF for a total process time of 2 min). Lysed cells were centrifuged at 38,000 x g for 45 min at 4°C. Clarified lysate was batch bound to 4mL of Ni-resin (Roche cOmplete His-Tag Purification Resin) for 1 h at 4°C with end-over-end rotation. Resin slurry was loaded on a plastic column and allowed to flow by gravity. Resin was sequentially washed with 40mL of PBS and 40mL of PBS with 20mM Imidazole (pH 8.0). Bound protein was eluted with PBS with 200mM Imidazole (pH 8.0) and elution collected in 5mL fractions. Protein concentration was determined by absorption at 280nm using a calculated extinction coefficient of 102010 M^{-1} cm^{-1}. Fractions with highest concentration were pooled and 20U of HRV-3C protease (Millipore-Sigma) were added. Cleavage reaction was subsequently dialyzed in 10 kDa MWCO cassette (Thermo-fisher Slide-A-Lyzer) overnight against 4L of buffer containing 25mM Tris-HCl (pH 7.5), 150mM NaCl and 5mM b-mercaptoethanol. Complete cleavage of MBP-6His from P-domain was confirmed by SDS-PAGE gel shift after Coomassie staining. Cleavage reaction was bound to 2mL of Ni-resin and incubated with end-over-end rotation for 1 h. Resin was loaded onto plastic column and flow-through containing cleaved P-domain was collected. Resin was washed with 5mL of dialysis buffer and both flow-throughs were pooled and concentrated to around 10mg/mL in 10kDa MWCO Amicon Ultra (Millipore-Sigma). Concentrated sample was loaded onto Superdex-200 Increase 10/300 GL (GE-healthcare) equilibrated with 25mM Tris-HCl (pH 7.5), 150mM NaCl and the peak corresponding to P-domain (elution volume 15.5mL) was collected. Purification was assessed by SDS-PAGE and purified P-domain was concentrated to 5mg/mL in 10kDa MWCO Amicon Ultra and stored at 4°C until further use.

Antibodies A1227 and A1431 were produced as previously described (McLellan et al., 2011). Briefly, heavy chain plasmids, containing the HRV3C cleavage site in the hinge region, and light chain plasmids were cotransfected in Expi293F cells (ThermoFisher) using a 3:1 ratio of Turbo293 transfection reagent (Speed Biosystem) to plasmid. Cells were incubated at 37°C, 5% CO_2, 130 rpm shaking, 70% humidity for 18 h and subsequently boosted with 80mL of AbBooster (Abi Scientific). Temperature was lowered to 33°C and expression was continued for additional 6 days. Cells were pelleted at 10000 x g, 20°C for 45 min and pellet discarded. Supernatant containing secreted antibody was supplemented with two tablets of protease inhibitors (Roche cOmplete EDTA-free). All subsequent steps were performed at 4°C. Cell supernantant was loaded by gravity onto 5ml of Protein A slurry (GE MabSelect) pre-equilibrated with PBS. Resin was washed with 50 mL of PBS and eluted in 1mL fractions with Pierce IgG Elution buffer. Elution was immediately neutralized with 1M Tris-Cl, pH 8.0 (final concentration 0.1M). Fractions with highest concentration (determined by A280 absorption) were pooled and 40U of HRV-3C protease (Millipore-Sigma) were added. Cleavage reaction was allowed to

proceed for 18 h at 4°C with end-over-end rotation. Cleavage reaction was assessed by SDS-PAGE before proceeding to next step. One mL of Ni-resin (Roche cOmplete His-Tag Purification Resin) loaded onto plastic column and overlaid with two mL of protein A resin. Ni-resin was used to capture His-tag, while protein A resin was used to capture free Fc. Both resins were equilibrated with PBS. Cleavage reaction was loaded by gravity onto resins and flow-through was collected. Resins were washed with 10mL of PBS and the second flow-through pooled with the first. Protein concentration was assessed by absorption at 280nm and removal of Fc and His-tag was verified after SDS-page and Coomassie staining. Purified Fab were concentrated to 10mg/mL in 10kDa MWCO Amicon Ultra (Millipore-Sigma) and purified on a Superdex-200 Increase 10/300 GL (GE-healthcare). Fractions containing pure Fab were pooled, concentrated to 10mg/mL in 10kDa MWCO Amicon Ultra (Millipore-Sigma) and stored at 4°C until further use.

Crystallization of Fab complex with P2002

Purified P2002 was mixed with 1.5-fold molar excess of purified Fab A1227 or Fab A1431 and incubated at 20°C for 1 h with end-over-end rotation. The mixture was injected onto Supredex 200 Increase 10/300 GL equilibrated with 25mM Tris pH 7.5, 150mM NaCl. Both complexes had a retention volume of around 12.3mL, consistent with two Fabs binding to one P-domain dimer. The fractions containing the complex were pooled and concentrated to 5mg/mL in MWCO 10kDa and immediately used for crystallization trials. The P2002-A1227 Fab complex was mixed with an equal volume of precipitant solution (10% polyethylene glycol 4000, 0.1M Tris pH 8.5, 50mM proline) and crystallized by sitting-drop vapor diffusion over the precipitant solution at 20°C. Rod-shaped crystals formed after 2 days. Crystals were harvested using Dual-Thickness MicroLoops (MiTeGen), cryoprotected using 30% ethylene glycol and immediately flash frozen in liquid nitrogen. The same procedure was used to crystallize the P2002-A1431 Fab complex, except the precipitant solution contained 10% polyethylene glycol 8000 and 0.1M CHES pH 9.5. Rod-shaped crystals formed after 2 days.

Structure determination and analysis

All diffraction data were collected at 100 K and 1.000 Å wavelength at the SER-CAT beamline ID-22 (Advanced Photon Source, Argonne National Laboratory) and processed with the HKL2000 suite (Otwinowski and Minor, 1997). A complete dataset was collected for the GII.4.2002 P domain-A1227 Fab complex to 2.6 Å using 0.25° oscillations and translating the crystal to different spots throughout data collection. A complete dataset was collected for the GII.4.2002 P domain-A1431 Fab complex to 3.1 Å from a single crystal using 0.5° oscillations. Each structure was solved by molecular replacement with the program PHASER (McCoy et al., 2007) using PDB ID 4OOV for the apo structure of GII.4.2002 P domain and PDB ID 3EYQ for the Fab as search models. Iterative cycles of model building and refinement were performed using COOT (Emsley and Cowtan, 2004) and PHENIX (Adams et al., 2004). Data collection and refinement statistics are shown in Table S2.

GI, GII and GII.4 sequence conservation scores were calculated using the AL2CO server (Pei and Grishin, 2001) and mapped onto the GII.4 P domain surface as described previously (Hansman et al., 2011). Buried surface area calculations and interface analyses were carried out with PISA (Krissinel and Henrick, 2007). Structure figures were prepared using the programs The PyMOL Molecular Graphics System, Version 2.0.5 Schrödinger, LLC and Chimera (Pettersen et al., 2004).

Neutralization Assay

Norovirus antibody neutralization

Serial dilutions of each mAb were prepared in complete medium without growth factors ($CMGF^{-}$) and pre-incubated with GII.P16-GII.4 Sydney stool filtrate (3.1×10^5 viral genomic copies). After 1 h incubation at 37°C and 5% CO_2, samples were diluted 1:2 in $CMGF^{-}$ supplemented with 1000 μM sodium glycochenodeoxycholate (GCDCA; Sigma-Aldrich) plus 100 μM ceramide (Santa Cruz Biotechnology). Duplicated, 100% confluent 4-day old differentiated monolayers, were inoculated. After 1 h incubation at 37°C and 5% CO_2, monolayers were washed twice with $CMGF^{-}$ and 100 μl of differentiation medium containing 500 μM GCDCA plus 50 μM ceramide was added to each well. For each set of infections, one plate was immediately frozen at -70°C and a duplicate plate was incubated at 37°C, 5% CO_2 for 24 h and frozen at -70°C.

For each monoclonal antibody, 4 independent experiments with 2 technical replicates for each mAb dilution on each experiment were performed. Neutralization was expressed as a measure of the reduction in viral genomic copies (as percentage) when compared to a control (no mAb) within each assay using real-time RT-qPCR.

Norovirus detection and quantification

Viral RNA was extracted from cultures (cells and media) at 1 h post-infection (hpi) and 24 hpi using the KingFisher instrument and MagMAX - 96 Viral RNA Isolation Kit (Applied Biosystems) according to the manufacturer's instructions. Norovirus RNA was detected by GI/GII TaqMan real-time RT-PCR (Cannon et al., 2017). Standard curves were generated using 10-fold serial dilutions of GII.4 Sydney RNA transcripts.

QUANTIFICATION AND STATISTICAL ANALYSIS

Antibody EC_{50} and IC_{50} were determined from log-transformed non-linear, dose-response sigmoidal curve fit data using GraphPad 7.02 (Lindesmith et al., 2015; Lindesmith et al., 2017). Antibody binding by EIA less than 0.2 OD at 2 μg/ml or blockade assays IC_{50} >2μg/ml was scored as negative for binding. Samples were tested in duplicate in a minimum of two independent experiments and the results reported in Figures 2 and 3.

DATA AND SOFTWARE AVAILABILITY

The datasets generated during the current study are available from the Lead Contact upon reasonable request. The accession numbers for the amino acids sequence of HuNoV strain capsids reported in this paper are GenBank: AAB50466.2 (GI.1), AFK75851.1 (GI.3), AFK75852.1 (GI.4), AFJ04707.1 (GII.4 1997), AFJ04708.1 (GII.4 2002), AFJ04709.1 (GII.4 2006b), AFV08795.1 (GII.4 2012), APA31970.1 (GII.4 2015), AFS33555.1 (GII.1), AAN08112.1 (GII.2), AFK75854.1 (GII.3), AAN05735.1 (GII.14), AKB94547.1 (GII.17), MK614455 (GII.4c), MK614084 (GII.4 2006a.581 virus-like particles), MK614085 (GII.4 2006a.581.A4A virus-like particles), MK614086 (GII.4 2006a.581.A4B virus-like particles), MK614081 (GII.4 1987. 2012A virus-like particles), MK614082 (GII.4 1987. 2012D virus-like particles), MK614083 (GII.4 1987. 2012E virus-like particles). The accession numbers for the atomic coordinates and structure factors for the reported crystal structures are Protein Data Bank (PDB): 6N81 and 6N8D.

Immunity, Volume *50*

Supplemental Information

Sera Antibody Repertoire Analyses Reveal Mechanisms of Broad and Pandemic Strain Neutralizing Responses after Human Norovirus Vaccination

Lisa C. Lindesmith, Jonathan R. McDaniel, Anita Changela, Raffaello Verardi, Scott A. Kerr, Veronica Costantini, Paul D. Brewer-Jensen, Michael L. Mallory, William N. Voss, Daniel R. Boutz, John J. Blazeck, Gregory C. Ippolito, Jan Vinje, Peter D. Kwong, George Georgiou, and Ralph S. Baric

Supplementary Figure Legends

Table S1, related to Figure 1. Description of monoclonal antibodies determined from Donors A, B, and C pre- and post-vaccination.

Clonotype ID	Representative CDR-H3 sequence	SHM (%)	IGHV usage	IGHJ usage	Pre-Vaccination		Post- Vaccination	
					Fraction (%)	XIC	Fraction (%)	XIC
A379	CARDYWNYHIRGWYPDYW	8.6	IGHV3-33	IGHJ4	58.28	5.E+08	31.67	1E+09
A6181	CARADGILVPPRAIWLDHW	12.0	IGHV3-33	IGHJ4	14.79	1.E+08	1.69	6E+07
A12910	CARGNRAVVPSHGFDSW	14.4	IGHV4-31	IGHJ4	6.43	5.E+07	1.67	6E+07
A101	CARSPRTVAIKPYYFDYW	16.7	IGHV3-53	IGHJ4	4.06	3.E+07	10.17	4E+08
A13567	CARLRASGYSYGYGFDHW	6.3	IGHV1-3	IGHJ4	3.96	3.E+07	1.36	5E+07
A1394	CARVPAAIRGVESW	5.2	IGHV1-2	IGHJ5	3.49	3.E+07	0.33	1E+07
A1431	CARDGPRPDGTGYAGPSNDYW	9.7	IGHV3-48	IGHJ4	1.73	1.E+07	10.32	4E+08
A9036	CARGVILARFDSW	4.4	IGHV4-31	IGHJ5	1.59	1.E+07		
A2088	CARLDTYGSGSYYDFW	6.7	IGHV5-51	IGHJ4	1.27	1.E+07		
A15154	CGRDVGPW	7.2	IGHV3-53	IGHJ5	1.13	9.E+06	0.29	1E+07
A11008	CARAGDSSGPW	1.1	IGHV4-34	IGHJ5	0.80	7.E+06	0.08	3E+06
A3865	CAREAIGVSGTTFDLW	17.4	IGHV1-46	IGHJ4	0.73	6.E+06		
A41497	CATLSTRAGGYFDKW	10.3	IGHV4-39	IGHJ4	0.36	3.E+06		
A11742	CARDPPATVNVW	3.3	IGHV3-53	IGHJ6	0.35	3.E+06	0.39	1E+07
A15504	CVRDAYGAFALFDYW	4.8	IGHV3-21	IGHJ4	0.34	3.E+06		
A11065	CARAKGTWGTPLLFW	11.8	IGHV1-69	IGHJ4	0.27	2.E+06		
A6211	CARALNYGDPTALNWFAPW	7.8	IGHV1-69	IGHJ5	0.18	1.E+06		
A1	CASRRRQDTSTVIRLNTMDDGFDVW	6.7	IGHV4-39	IGHJ3	0.12	1.E+06	0.19	7E+06
A12246	CAREGSLRYFDWSRGGTPGYFDYW	1.4	IGHV4-34	IGHJ4	0.10	8.E+05		
A5920	CTRDQCGGDCSDFW	9.3	IGHV3-74	IGHJ4			9.98	4E+08
A834	CARAVPLLGELSIRLSTFDNW	7.8	IGHV4-39	IGHJ4			5.19	2E+08
A4731	CARSRVPSGYCDSVSCYRSYGMDLW	8.9	IGHV1-69	IGHJ6			3.58	1E+08
A16474	CAGRASGSSPPHW	7.9	IGHV3-23	IGHJ4			3.18	1E+08
A16363	CAGHRGYSSGWPLDYW	11.5	IGHV4-34	IGHJ4			2.90	1E+08
A468	CARDRPLASGYDRDDYYGMDVW	7.9	IGHV4-31	IGHJ6			2.26	8E+07
A3612	CARVVAGYYSGSQRYSRSAVIDHW	8.6	IGHV4-59	IGHJ4			1.74	6E+07
A22288	CARAGIIHAINGFDIW	5.8	IGHV1-2	IGHJ3			1.68	6E+07
A10667	CAKGGFDQVPNRENIGGTLLGNW	6.5	IGHV3-23	IGHJ4			1.28	5E+07
A12994	CARGRFLAPFDYW	1.5	IGHV4-34	IGHJ4			1.08	4E+07
A1227	CAKDKTRTLRLGYSGMDVW	8.0	IGHV3-23	IGHJ6			0.90	3E+07
A3804	CARDGALRYDSTGNHYGSW	8.9	IGHV1-2	IGHJ4			0.84	3E+07
A2705	CAKSHYGDTYDSESTYYFDLW	8.8	IGHV1-69	IGHJ2			0.74	3E+07
A3423	CARGTIYDFLAGDRGLGFW	9.3	IGHV1-18	IGHJ4			0.64	2E+07
A4965	CAKCRPGIAAAGPDYW	3.0	IGHV3-30	IGHJ4			0.64	2E+07
A1945	CARGGDYADPQFQRW	5.8	IGHV4-34	IGHJ1			0.52	2E+07
A7704	CVKDRGRRLGSLYPQFGSW	10.5	IGHV3-23	IGHJ4			0.49	2E+07
A2188	CAREGRRDGRPSAVDYW	0.7	IGHV1-2	IGHJ4			0.44	2E+07
A577	CARGSYGDTFGLHPW	8.2	IGHV4-59	IGHJ5			0.43	2E+07
A46215	CATGRVSSNWYRHEYIHYW	7.1	IGHV4-31	IGHJ1			0.40	1E+07
A44984	CARFPSAGYGMDVW	7.6	IGHV3-23	IGHJ6			0.34	1E+07
A4881	CGRWRDGAGAVDNW	7.5	IGHV3-73	IGHJ4			0.33	1E+07
A1438	CARNGEPVYNSWDGNRNHAFDPW	7.8	IGHV4-34	IGHJ3			0.33	1E+07
A5063	CALVTVTRGPVFDYW	5.7	IGHV4-59	IGHJ4			0.28	1E+07
A12572	CARGAYYSDSSGYYKYFHYW	2.6	IGHV3-48	IGHJ1			0.25	9E+06
A3769	CARAGYGSGSYYKHFYYGMDVW	8.5	IGHV5-51	IGHJ6			0.22	8E+06
A2405	CARQQRQSDTSAYYGYW	8.3	IGHV5-51	IGHJ4			0.19	7E+06
A1698	CARGIAARQWYFDLW	4.7	IGHV4-31	IGHJ2			0.19	7E+06
A11278	CARDAGYSSSSENRAPMDW	1.5	IGHV1-2	IGHJ4			0.18	6E+06
A45505	CAQFGSPGLAATLSYFGSW	10.3	IGHV3-23	IGHJ4			0.16	6E+06
A14683	CARYVQGAFDIW	3.0	IGHV4-39	IGHJ3			0.12	4E+06
A28571	CAREKTVSGTDWFDYW	10.8	IGHV4-4	IGHJ4			0.10	4E+06
A14002	CARRVFDHSEYRVAEDKW	9.3	IGHV1-18	IGHJ4			0.08	3E+06
A10111	CVRGIASSVSPFWYFDLW	14.7	IGHV4-39	IGHJ2			0.07	2E+06
A31504	CARGPEKYYDWLGYFDSW	6.3	IGHV4-31	IGHJ4			0.05	2E+06
A3717	CAKDHEVGAPSDLFDYW	6.7	IGHV3-33	IGHJ4			0.03	1E+06
A3439	CARETPGVSDPW	2.5	IGHV4-31	IGHJ5			0.01	4E+05

Clonotype ID	Representative CDR-H3 sequence	SHM (%)	IGHV usage	IGHJ usage	Pre-Vaccination		Post- Vaccination	
					Fraction (%)	XIC	Fraction (%)	XIC
B13309	CAKLDYYDPW	7.6	IGHV3-30	IGHJ5	85.6	1.E+08	13.10	2E+08
B10310	CAKDSGLARSLPDYW	4.0	IGHV3-9	IGHJ4	4.20	5.E+06		
B3312	CARRDGVLDW	1.2	IGHV1-46	IGHJ4	3.12	4.E+06		
B9951	CSRDVARARRFGELRTLSDYASLPSSKPFDPW	13.4	IGHV3-49	IGHJ5	3.00	4.E+06		
B6525	CARAGGSGTYYPNWFDPW	7.2	IGHV4-39	IGHJ5	2.88	3.E+06		
B5394	CARALFSNYYDNRGYYHELGHW	8.6	IGHV3-30	IGHJ4	1.24	1.E+06		
B10587	CAKVRGRVGGATFYHGMDVW	10.3	IGHV3-23	IGHJ6			47.50	6E+08
B231	CARLKSAYYFDSW	12.5	IGHV1-2	IGHJ4			8.23	1E+08
B401	CSRGKRRVAGPTFYSGMDVW	11.8	IGHV3-23	IGHJ6			6.77	9E+07
B137	CARMASGYYDWW	7.1	IGHV1-2	IGHJ4			5.15	7E+07
B12268	CARVLETNWDPNWFDRW	7.1	IGHV4-31	IGHJ5			4.17	6E+07
B403	CARGEGGGTLPFEYW	9.2	IGHV4-31	IGHJ4			3.97	5E+07
B3742	CARHFGSGSGSYSRTVQSW	4.3	IGHV4-39	IGHJ5			2.29	3E+07
B404	CAKGKRRVGGAAFYSGLDVW	9.0	IGHV3-23	IGHJ6			2.00	3E+07
B6907	CARVMEQYYDSRAMDHW	8.2	IGHV4-31	IGHJ4			1.15	2E+07
B4651	CARFDCFSTTCRHYYAMDVW	7.7	IGHV4-39	IGHJ6			1.02	1E+07
B5944	CARDFSDW	4.4	IGHV4-39	IGHJ4			0.99	1E+07
B68	CARDRGGRDLDTW	8.1	IGHV3-33	IGHJ5			0.97	1E+07
B542	CARLLYDRSGYYYFDSW	4.9	IGHV4-39	IGHJ4			0.92	1E+07
B1021	CARDPGGVLVAGLFHRNDVCDIW	10.9	IGHV1-3	IGHJ3			0.80	1E+07
B14504	CARDRGFYGVDVW	13.2	IGHV1-3	IGHJ6			0.57	8E+06
B980	CARVGLMGGSTPFDYW	3.6	IGHV4-31	IGHJ4			0.16	2E+06
B11062	CAREVSTGACGYW	0.0	IGHV3-7	IGHJ4			0.14	2E+06
B3645	CARDDVRYGFMVYW	8.7	IGHV1-18	IGHJ4			0.11	1E+06
B44	CARGGYGANYGDHW	10.9	IGHV7-81	IGHJ4			0.01	1E+05
C601	CARAAPLQWELKPGPFNSW	7.5	IGHV3-53	IGHJ4	70.36	8E+08	91.92	1E+10
C16720	CSRIGFLQWSRSNDAFDVW	15.1	IGHV4-55	IGHJ3	8.17	9E+07	2.05	3E+08
C46	CARDLKFGVGVPLTPYYDSW	10.8	IGHV3-11	IGHJ4	7.21	8E+07	0.75	9E+07
C26886	CAKDRNTNRDGLGNHFDSW	8.9	IGHV1-2	IGHJ5	6.05	7E+07	1.38	2E+08
C1768	CARLRVGSSSGRELDYW	4.0	IGHV5-51	IGHJ4	3.80	4E+07	0.23	3E+07
C159	CAKVAGTSFYYFYAMDVW	4.3	IGHV3-30	IGHJ6	1.36	2E+07	0.05	6E+06
C21286	CARPAAGALRTGDYSYGFDPW	15.0	IGHV1-18	IGHJ5	1.17	1E+07		
C10582	CARGNQGVELRKFWFDPW	11.9	IGHV4-59	IGHJ5	0.69	8E+06	0.49	6E+07
C13227	CARWDPYDFW	13.3	IGHV1-3	IGHJ4	0.42	5E+06		
C12556	CARGPRRIMHYYDNRGPIDFW	12.2	IGHV3-30	IGHJ4	0.38	4E+06		
C9325	CARRGVRIPLDAFDVW	12.8	IGHV4-39	IGHJ3	0.37	4E+06		
C602	CARGRSGWETRPHYFDYW	7.9	IGHV3-49	IGHJ4			1.11	1E+08
C12468	CARGGWSMDVW	6.6	IGHV4-59	IGHJ6			0.65	8E+07
C380	CAKAKGDYYESSGYLFDSW	3.4	IGHV3-23	IGHJ4			0.54	7E+07
C12232	CAREGGGYFYGPGNHYRYYYAMDVW	10.8	IGHV4-59	IGHJ6			0.27	3E+07
C9502	CARVSRTTWGFGYW	4.4	IGHV4-31	IGHJ4			0.17	2E+07
C39728	CARGRPPNWELFVDYFDYW	8.4	IGHV3-53	IGHJ4			0.16	2E+07
C6265	CARVFLDYEIRTGYYTQYNFDYW	9.5	IGHV4-39	IGHJ4			0.10	1E+07
C2873	CARSPPNTNYLRDAFDIW	13.4	IGHV4-59	IGHJ3			0.09	1E+07
C7027	CARRGSRTAASYGMDVW	0.8	IGHV1-2	IGHJ6			0.03	4E+06

Table S2, related to Figures 4 and 5. Crystallographic data collection and refinement statistics.

	GII.4.2002+A1431	GII.4.2002+A1227
PDB ID	6N8D	6N81
**Data collection**		
Space group	C 2	$P\ 2_1$
Cell dimensions		
a, *b*, *c* (Å)	147.4, 157.4, 110.4	82.6, 150.1, 117.3
α, β, γ (°)	90, 112.9, 90	90, 95.1, 90
Resolution (Å)	50 - 3.1 (3.15-3.10)*	50 – 2.6 (2.64-2.60)
Unique reflections	39748 (2068)	84782 (4171)
R_{merge}	13.6 (87.7)	12.0 (45.3)
R_{pim}	8.5 (55.4)	7.8 (30.7)
$I / \sigma I$	15.7 (2.2)	12.3 (2.1)
Completeness (%)	94.9 (97.4)	96.0 (95.5)
Redundancy	3.4 (3.4)	3.2 (2.8)
Wilson B-factors ($Å^2$)	61	31
**Refinement**		
Resolution (Å)	41.62 – 3.10 (3.21-3.10)	46.09 - 2.58 (2.67 – 2.58)
No. reflections	39673 (3942)	84760 (7381)
R_{work} / R_{free}	0.18 (0.26) / 0.23 (0.33)	0.17 (0.24) / 0.21 (0.30)
No. atoms	11214	11539
Protein	11214	11232
Water	0	307
B-factors ($Å^2$)		
P domain	49	38
Fab	109	54
Water	-	39
R.m.s. deviations		
Bond lengths (Å)	0.007	0.003
Bond angles (°)	1.26	0.78
Ramachandran statistics		
Favored (%)	92	96
Outliers (%)	0.6	0.3

One crystal was used for each structure

* Values in parentheses are for highest-resolution shell

Table S3, related to STAR methods. Primers for amplifying multiplex VH repertoire.

V_H	Primer (5'->3')
VH1-fwd	CAGGTCCAGCTKGTRCAGTCTGG
VH157-fwd	CAGGTGCAGCTGGTGSARTCTGG
VH2-fwd	CAGRTCACCTTGAAGGAGTCTG
VH3-fwd	GAGGTGCAGCTGKTGGAGWCY
VH4-fwd	CAGGTGCAGCTGCAGGAGTCSG
VH4-DP63-fwd	CAGGTGCAGCTACAGCAGTGGG
VH6-fwd	CAGGTACAGCTGCAGCAGTCA
VH3N-fwd	TCAACACAACGGTTCCCAGTTA
IgM-rev	GGTTGGGGCGGATGCACTCC
IgG-all-rev	SGATGGGCCCTTGGTGGARGC
IgA-all-rev	GGCTCCTGGGGGAAGAAGCC

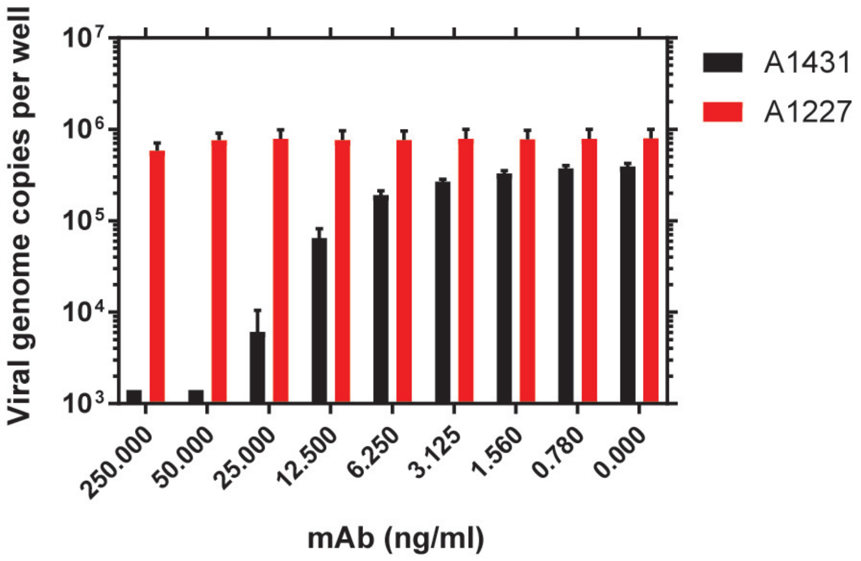

Figure S1, related to Figure 2. A1431 neutralizes GII.P16-GII.4 Sydney strain.

Viral genome copies per well at 24hs post-infection as a function of antibody concentration. Error bars represent SEM. Samples were tested in duplicate in four independent experiments.

a

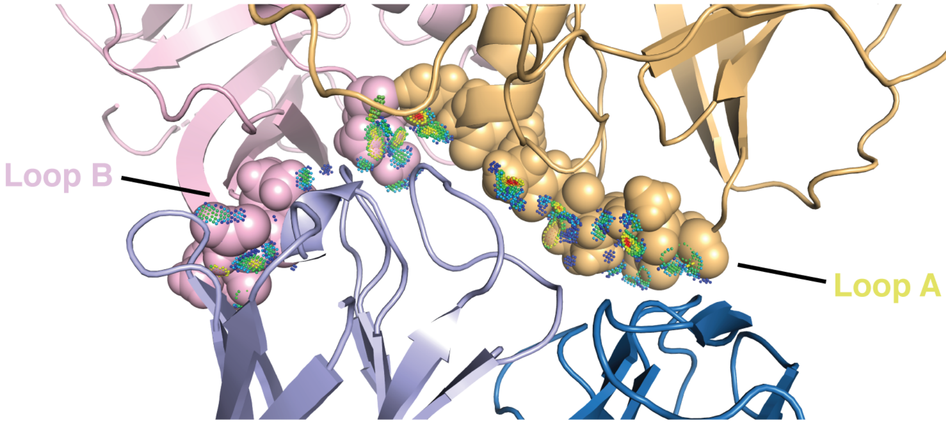

b

```
         448       458       468       478       488       498
GII.4    NLDCLLPQEWVQHFYQEAAPAQSDVALLRFVNPDTGRVLFECKLHKSGYVTVAHT
GII.14   AIDCLLPQEWIEHFYQEAAPSQSDIALVRFINPDTGRVLLEAKLHKQGFLTVAAS
GII.3    VLDCLVPQEWVQHFYQESAPAQTQVALVRYVNPDTGRVLFEAKLHKLGFMTIAKN
GII.17   IIDCLIPQEWIQHFYQESAPSQSDVALIRYVNPDTGRTLFEAKLHRSGYITVAHS
GII.2    AIDCLLPQEWVQHFYQEAAPSMSEVALVRYINPDTGRALFEAKLHRAGFMTVSSN
GII.1    AIDCLLPQEWIQHFYQESAPSPTDVALIRYTNPDTGRVLFEAKLHRQGFITVANS
GI.3     SVPCTIPQEFVTHFVNEQAPTRGEAALLHYLDPDTHRNLGEFKLYPEGFMTCVPN
GI.1     NLPCLLPQEYISHLASEQAPTVGEAALLHYVDPDTGRNLGEFKAYPDGFLTCVPN
G1.4     LVPCLLPQEYITHFISEQAPIQGEAALLHYVDPDTNRNLGEFKLYPGGYLTCVPN
                         Loop A            Loop B
```

Figure S2, related to Figure 4. A1227 interacts with the GII.4.2002 P domain. **a**) Two main loops in P2002 are responsible for interaction with A1227. Loop A (orange spheres) is composed of 7 amino acids (Q463-Q469) and is located at the C-terminus of the A monomer. Loop B (pink spheres) is composed of 3 amino acids (D481-G483) and is located at the C-terminus of monomer B. van der Waals (vdW) interactions were calculated using Protein-Interaction-Viewer software installed as Pymol plugin (Word et al., 1999), which implements the small-probe contact dot surfaces technique. Dot colors indicate the gap distance between vdW radii at each dot position (using a 0.25 Å probe): green or yellow for good contact (greens for narrow gaps, yellows for slight overlaps <0.2 Å), blues for wider gaps (>0.25 Å), orange or red spikes for small interpenetrations. **b**) Sequence alignment of several GI and GII genotypes highlighting degree of conservation among interacting residues (Deep purple denotes identical residues).

```
GII.4.2002        AHQNEPQQWVLPSYSGRTGHNVHLAPAVAPTFPGEQLLFFRSTMPGCSGYPNM-NLDCLL   453
GII.4.1987        HHQNEPQQWVLPNYSGRTGHNVHLAPAVAPTFPGEQLLFFRSTMPGCSGYPNM-NLDCLL   452
GII.4.1997        NHQNEPQQWVLPNYSGRTGHNVHLAPAVAPTFPGEQLLFFRSTMPGCSGYPNM-NLDCLL   452
GII.4.2006a       THQNEPQQWELPDYSGRGIHNVHLAPAVAPTYPGEQILFFRSTMPGCSGYPNM-NLDCLL   453
GII.4.2006b       THRNEPQQWVLPSYSGRNVHNVHLAPAVAPTFPGEQLLFFRSTMPGCSGYPNM-DLDCLL   453
GII.4.2012        THRNEPQQWVLPSYSGRNTHNVHLAPAVAPTFPGEQLLFFRSTMPGCSGYPNM-DLDCLL   453
GII.4.2015        THRNEPQQWVLPSYSGRNTHNVHLAPAVAPTFPGEQLLFFRSTMPGCSGYPNM-DLDCLL   453
GI.1              PSGSQVDLWKIPNYGSSITEATHLAPSVYPPGFGEVLVFFMSKMPGPGAY----NLPCLL   437
GI.3              GHRGSVDPWVIPRYGSTLTEAAQLAPPIYPPGFGEAIVFFMSDFPIAHGA-NGLSVPCTI   452
GI.4              SGGANTNFWKIPDYGSSLAEASQLAPAVYPPGFNEVIVYFMASIPGPNQSGSPNLVPCLL   451
GII.1             ----HFDQWALPSYSGRLTLNMNLAPSVSPLFPGEQLLFFRSHIPLKGGTSDG-AIDCLL   448
GII.2             ----HFNQWVVPRYAGALNLNTNLAPSVAPVFPGERLLFFRSYIPLKGGYGNP-AIDCLL   455
GII.3             --EAEFQQWSLPNYSGQFTHNMNLAPAVAPNFPGEQLLFFRSQLPSSGGWSNG-VLDCLV   461
GII.14            --QHPFRQWSLPNYGGHLALNNHLAPAVTPLFPGEQILFFRSHIPSAGGHTDG-AIDCLL   449
GII.17            -DGHPFRQWELPNYSGELTLNMNLAPPVAPNFPGEQLLFFRSFVPCSGGYNQG-IIDCLI   453
                          * :* *..      :*** : *   .* :::* : .*          : * :

GII.4.2002        PQEWVQHFYQEAAPAQSDVALLRFVNPDTGRVLFECKLHKSGYVTVAHT--GQHDLVIPP   511
GII.4.1987        PQEWVLHFYQEAAPAQSDVALLRFVNPDTGRVLFECKLHKSGYITVAHT--GPYDLVIPP   510
GII.4.1997        PQEWVQHFYQEAAPAQSDVALLRFVNPDTGRVLFECKLHKSGYVTVAHT--GPHDLVIPP   510
GII.4.2006a       PQDWVRHFYQEAAPAQSDVALLRFVNPDTGRVLFECKLHKSGYVTVAHT--GQHDLVIPP   511
GII.4.2006b       PQEWVQHFYQEAAPAQSDVALLRFVNPDTGRVLFECKLHKSGYVTVAHT--GQHDLVIPP   511
GII.4.2012        PQEWVQYFYQEAAPAQSDVALLRFVNPDTGRVLFECKLHKSGYVTVAHT--GQHDLVIPP   511
GII.4.2015        PQEWVQYFYQEAAPAQSDVALLRFVNPDTGRVLFECKLHKSGYVTVAHT--GQHDLVIPP   511
GI.1              PQEYISHLASEQAPTVGEAALLHYVDPDTGRNLGEFKAYPDGFLTCVPNGASSGPQQLPI   497
GI.3              PQEFVTHFVNEQAPTRGEAALLHYLDPDTHRNLGEFKLYPEGFMTCVPNSSGTGPQTLPI   512
GI.4              PQEYITHFISEQAPIQGEAALLHYVDPDTNRNLGEFKLYPGGYLTCVPNSSSTGPQQLPL   511
GII.1             PQEWIQHFYQESAPSPTDVALIRYTNPDTGRVLFEAKLHRQGFITVANS--GSRPIVVPP   506
GII.2             PQEWVQHFYQEAAPSMSEVALVRYINPDTGRALFEAKLHRAGFVTVSSN--TSAPVVVPA   513
GII.3             PQEWVQHFYQESAPAQTQVALVRYVNPDTGRVLFEAKLHKLGFMTIAKN--GDSPITVPP   519
GII.14            PQEWIEHFYQEAAPSQSDIALVRFINPDTGRVLLEAKLHKQGFLTVAAS--GDHPIVMPT   507
GII.17            PQEWIQHFYQESAPSQSDVALIRYVNPDTGRTLFEAKLHRSGYITVAHS--GDYPLVVPA   511
                  **::: :: .* **   : **::: :*** * * * * :  *::*   .        :*
```

Figure S3, related to Figure 5. A ClustalW alignment of GI and GII P-domain sequences shows residues that directly interact with A1431 are conserved among GII.4 strains but differ in other GI and GII strains. Consensus symbols indicate varying degrees of conservation at each position: Fully conserved (*), strong (:) and weak (.) level of conservation. Residues involved in binding A1431 Fab are highlighted in blue (see **Fig. 5c** for interaction with A1431 and **Fig. 5d** for location of these residues on the P domain dimer).
